# Odor Experience Stabilizes Glomerular Output Representations in Two Mouse Models of Autism

**DOI:** 10.1523/ENEURO.0271-25.2025

**Published:** 2025-10-28

**Authors:** Kassandra L. Sturm, Daryna Semak, Zoe A. Scheier, Raddy L. Ramos, Gonzalo H. Otazu

**Affiliations:** College of Osteopathic Medicine, New York Institute of Technology, Old Westbury, New York 11568

**Keywords:** autism, calcium imaging, Cntnap2, olfaction, Shank3

## Abstract

Novel stimuli can be stressful for individuals with autism spectrum disorders (ASD), though repeated exposure can reduce this effect. In *Cntnap2^-/-^* and *Shank3B^+/−^* mouse models of ASD, novel background odors impaired behavioral target odor recognition but that deficit improved with training. To investigate the neural basis of this improvement, we used wide-field calcium imaging to measure olfactory bulb responses in *Cntnap2^−/−^* and *Shank3B^+/−^* mice and WT mice of either sex. Training with background odors enhanced both behavioral performance and neural discriminability of odor mixtures in both *Cntnap2^−/−^* and *Shank3B^+/−^* as well as WT mice. Naive *Cntnap2^−/−^* and *Shank3B^+/−^* mice showed greater trial-to-trial neural variability than WT mice, but training stabilized neural responses. Critically, training produced a widespread reduction in olfactory bulb responses to background odors in ASD models, but not in WT mice. Thus, despite similar behavioral improvements as WT mice, *Cntnap2^−/−^* and *Shank3B^+/−^* mice relied on a distinct broad suppression of background odor responses to enhance olfactory coding in the presence of background odors.

## Significance Statement

Abnormal sensory responses to unfamiliar stimuli are a hallmark of autism spectrum disorders (ASD) and can be alleviated with prolonged exposure. Neural variability in sensory responses is observed in both individuals with ASD and different mouse models, but its impact on behavior remains unclear. Using two ASD-associated gene mutation models (*Shank3* and *Cntnap2*), we performed wide-field calcium imaging in the olfactory bulb. Prolonged exposure to a background odor stabilized olfactory bulb activity, enhancing neural coding and discrimination in these two different mouse models of ASD, but not in WT mice, despite similar improvements in behavior. This work highlights how neural activity fluctuations in the olfactory bulb influence behavior in ASD, offering insights into sensory processing mechanisms and potential therapeutic strategies.

## Introduction

People with autism have a striking adherence to routine and sensory patterns that when disrupted can cause extreme distress. This characteristic is described as an “insistence on sameness” in the DSM-V (Rosen et al., 2021). The DSM-V also includes hyperreactivity to sensory input as part of the definition of ASD. Abnormal behavioral responses to novel sensory stimuli in ASD can be ameliorated after long exposure (Storch et al., 2020).

The “insistence on sameness” in ASD is also prominent in the eating habits of autistic children with restricted diets (Mayes and Zickgraf, 2019) composed of a narrow set of food items compared with the broader repertoire of typically developing children (Bandini et al., 2010). Notably, food repertoire could be expanded by gradually exposing the autistic child over weeks to different aspects of new food items including smell due to the significant role olfactory deficits play in diet restriction (Ernsperger and Stegen-Hanson 2004; Nadon et al., 2011; Luisier et al., 2019). Thus, novel food smells affect autistic children’s behavior compared with typically developing children. However, the effects of novel odors in autistic children are not fixed and can be ameliorated with prolonged experience with an odor (Scheier et al., 2024).

The neural basis of sensory processing deficits in ASD are studied using mouse models of ASD, which can be created by introducing mutations in different genes that have been associated with ASD (Kazdoba et al., 2016). Despite the diversity of functions of these ASD-associated genes, the novelty of an odor also affects the behavioral responses in multiple mouse models of autism compared with WT mice. The Fmr1 knock-out (KO) mouse model of fragile X syndrome (The Dutch-Belgian Fragile X Consortium, 1994) has deficits in learning to discriminate difficult novel odor mixtures compared with WT mice (Kuruppath et al., 2023). The *Tbr1^+/−^* mice (Bulfone et al., 1998) do not explore novel odors compared with WT mice (Huang et al., 2019). Novel background odors cause deficits in target identification in *Shank3B^−/+^* (Peça et al., 2011) and *Cntnap2^−/−^* mice (Poliak et al., 2003) compared with WT mice. *Shank3B^−/+^* and *Cntnap2^−/−^* mice behavior improves with prolonged exposure to the background odor eventually matching the behavior of WT mice (Li et al., 2023; Ryndych et al., 2023).

Rodent models of autism spectrum disorder (ASD) exhibit increased variability in sensory-evoked neural responses across multiple brain regions. For instance, heightened variability has been reported in the somatosensory cortex (Bhaskaran et al., 2023) and auditory cortex (Scott et al., 2022). Similar effects are observed in the olfactory bulb, where ASD mouse models show more variable odor-evoked responses compared with WT mice. In anesthetized *Shank3B^−/−^* and *Cntnap2^−/−^* mice, odor-evoked activity in olfactory bulb neurons is significantly more variable than in WT mice (Geramita et al., 2020). Likewise, mitral cells in the Fmr1 knock-out (KO) model of fragile X syndrome exhibit unstable membrane potentials and increased response variability following electrical stimulation of the glomeruli (Kuruppath et al., 2023). Increased variability is also seen in awake *Shank3B^−/+^* mice, which display greater fluctuations in intrinsic signal responses to passive odor exposure compared with WT animals (Ryndych et al., 2023). Together, these findings suggest that elevated neural variability is a shared feature across ASD models and sensory modalities, making them a powerful tool for dissecting the circuit-level mechanisms underlying sensory processing deficits in autism.

We do not know whether prolonged exposure to an odor can stabilize olfactory bulb responses and subsequently enhance olfactory-guided behaviors in mouse models of ASD. Here we show that extensive training with a background odor reduced olfactory bulb evoked activity and variability, permitting the identification of the embedded target odors in *Shank3B^−/+^* and *Cntnap2^−/−^* mice. These results in two distinct mouse models of ASD points toward instability in the olfactory bulb as a common circuit mechanism for deficits in olfactory discrimination in ASD.

## Materials and Methods

### Animals

We have crossed the *Cntnap2* homozygous mouse model of autism (Poliak et al., 2003; JAX Stock No: 017482) and the *Shank3B* heterozygous (Peça et al., 2011; JAX Stock No: 017688) with the C57BL/6J-Tg(Thy1-GCaMP6f) GP5.11Dkim/J. (JAX Stock No: 024339). Pups were genotyped until we obtained *Shank3B^+/−^* Thy1-GCaMP6f and *Cntnap2^−/−^* Thy1-GCaMP6f mice (see table genotype and age of mice). Mice of either sex were used. We have used the C57BL/6J-Tg(Thy1-GCaMP6f) GP5.11Dkim/J as WT controls.

### Surgical procedure

The surgical procedure was modified from previously published method (Li et al., 2023). Mice between 4 and 10 months of age, 20–25 g, were anesthetized using ketamine/xylazine (KX, initial dose 70/7 mg/kg), further supplemented as needed to keep the pedal withdrawal reflex diminished. Respiration and lack of pain reflexes were monitored throughout the experiment. Ophthalmic ointment was applied to the eyes. Aseptic technique was used, first clipping hair and prepping with betadine on the skin. Lidocaine and iodine were applied topically to skin (as analgesic and antiseptic, respectively). After the animals were deeply anesthetized, they were mounted in a stereotaxic frame with ear bars. A small incision (2−3 cm) was made into the skin above the surgical site. A titanium head bar was cemented on the skull near the lambda suture using light-cured Vitrobond (3 M) further reinforced with dental acrylic. We also modified the window implant procedure from (Otazu et al., 2015) and used skull thinning instead of cranial windows to get more stable recordings. The bone over the olfactory was thinned using a dental drill. As the vasculature became visible, a droplet of Vetbond was applied over the bone and let dry for 5 min. After that, a drop of cyanoacrylate glue (Pacer Technologies, catalog #ZAP-A-GAP CA+) Zap-a-Gap was applied over the skull, and a 3.5-mm-diameter glass coverslip was pressed against the skull for 10 min. The coverslip borders were reinforced with dental acrylic. Animals were allowed to recover for 1 week before starting water deprivation. During the behavioral sessions, the animal’s heads were held firmly in place by mounting the titanium head bar onto a custom-built holder.

### Behavior

Detailed training protocols were previously described (Li et al., 2023). Animals were deprived of water for 7 d before starting their training. Weight was maintained at 80–85% of their weights after recovering for 1 week after the surgery. Each day before their task, animals were head fixed, and their coverslips were cleaned. Excitation light was adjusted until clear respiration events could be seen synchronized with respiration in the fluorescent signal.

### Image acquisition

Imaging setup was similar to the previously described method (Li et al., 2023). Briefly, wide-field fluorescence imaging of the olfactory bulb was done using a pair of back-to-back SLR lenses with a 50 mm f/1.4 lens (Nikon) used as objective and second lens Tamron AF 90 mm f/2.8 Di SP AF/MF 1:1 Macro Lens coupled to an sCMOS camera (CS2100 M, Thorlabs). The camera was fitted with a low-pass filter with cut-on wavelength of 500 nm (FELH0500, Thorlabs). Pixels were binned by software in a 4 by 4 square, resulting in a resolution of 13.2 µm per pixel. Images were acquired at a rate of 20 Hz. We used a blue 470 nm LED (M470L4, Thorlabs) mounted with a GFP excitation filter (MF469-35, Thorlabs) and a diffuser (ACL2520U-DG6-A, Thorlabs) to produce uniform and pattern free illumination. The illumination was set up to the minimum value that produced noticeable increases in fluorescent synchronized with respiration. The aperture of the objective lens was opened to the maximum value of f/1.4 to maximize light collection. Image acquisition started 7 s before odor onset on each trial. Image acquisition was done using ThorCam subroutines called from Matlab. We used an airflow sensor (1000 SCCM AWM300V, Honeywell) circuit (Bolding and Franks, 2018) as previously described (Li et al., 2023).

### Image analysis

Image analysis was done using Matlab ver 2017. Images recorded on each day were registered to a common image using the imregconfig Matlab function with the (“multimodal”) setting. A normalized signal df/*f*_0_ was calculated for each pixel for the average image using as *f*_0_ the average response of the 7 s period preceding odor onset. In order to remove the broad spatial signal in response to odors, the df/*f*_0_ were convolved with a Gaussian of radius *σ* = 199 µm and this low-pass filtered signal was subtracted from the original df/*f*_0_. To remove high spatial frequency spatial noise, the resulting images were further convolved with a Gaussian of radius *σ* = 39 µm. An average was calculated over all the odor presentations with the same odor combinations. ROIs were drawn manually using ImageJ (Schindelin et al., 2015) over activated glomeruli using the average images of known background odors, as well as the df/*f*_0_ images of the individual odor presentations with known background odors.

We quantified glomerular activation as the mean value of the *z*-score across all selected pixels in an ROI. To normalize the signal of each ROI, a *z*-score was calculated for each ROI using the values of df/*f*_0_ of the 7 s of air, before the onset of the odor period to calculate the mean and the standard deviation.

## Results

**Figure 1.**
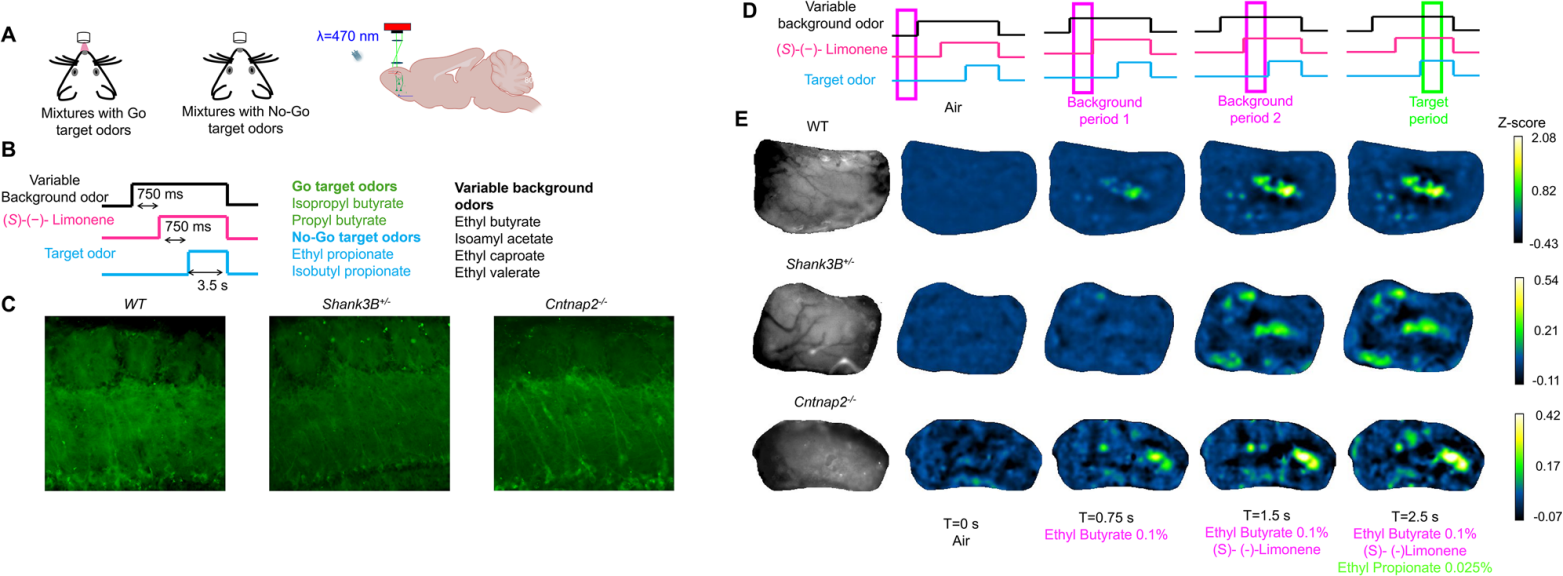
Wide-field calcium imaging of the olfactory bulb neural activity in the *Shank3B^−/+^* and the *Cntnap2*^−/−^ mice during target recognition in background odors. ***A***, Wide-field imaging was used to measure the glomerular responses in the olfactory bulb through thinned bone in water-deprived mice. Mice were trained to lick for odor mixtures that contained go target odor to receive water and to refrain from licking for odor mixtures that contained no-go target odors. ***B***, Mice were trained to identify target odors embedded in a set a variable background odors and a fixed background odor (limonene). ***C***, *Shank3B^−/+^* and the *Cntnap2*^−/−^ mice were crossed to Thy1-GCaMP6f mice that expressed GCaMP6f in mitral and tufted cells as seen in histological section, with similar expression to the WT Thy1-GCaMP6f line. ***D***, ***E***, Examples of average glomerular responses over ∼10 repeats of correct rejection in response with ethyl butyrate and (s)-(−)limonene at 0.1% of saturated vapor pressure as backgrounds, with the no-go target odor being ethyl propionate at 0.025% of saturated vapor pressure. Images are averaged over 750 ms time windows. Glomerular responses were seen in all three genotypes (see attached videos). The fluorescence signal reflects the activity in cells in the olfactory bulb and not axonal projections from other parts of the brain (Extended Data Fig. 1-1). The sex and ages of the animals used are on the table on Extended Data Figure 1-2.

To investigate the neural mechanisms in the olfactory bulb that support odor detection in autism mouse models, we recorded olfactory bulb responses in *Cntnap2*^−/−^ and *Shank3B^−/+^* (Fig. 1*A*,*B*) mice before and after training to detect weak target odors (0.025% vapor saturation) presented alongside strong background odors (0.1% vapor saturation). We compared these responses to those of wild-type (WT) mice to identify changes specific to mouse models of ASD associated with learning.

10.1523/ENEURO.0271-25.2025.f1-1 Figure 1-1 **Widefield fluorescence reflects activity from neurons in the olfactory bulb. A.** We injected AAV virus to express GCaMP6f in the piriform cortex of a *Shank3B^+/-^* mouse. After two weeks, we imaged the axons using thinned bone technique as we used with the Thy1-GCaMP6f mice. **B.** Histology section showing strong expression of GCaMP6f mice in axons from piriform cortex in the olfactory bulb. **C-D.** Examples of widefield images in response to odors did not reveal glomerular structures in the virus injected mouse, but they were evident in the Thy1-GCaMP6f mouse. **E-F.** GCaMP6f is expressed in the soma and the dendrites of mitral and tufted cells (see Figure 1C), so widefield calcium signal could potentially reflect activity from these two cell populations. Two-photon microscopy has revealed that mitral cells somatic calcium responses in WT mice in response to pure go-target odors (S^+^ stimulus) are suppressed by reward association, whereas calcium responses in the more superficial tufted cells do not. In addition, calcium signals from mitral cell apical dendrites are not suppressed by reward association (Lindeman et al. 2024). To determine whether the widefield calcium signal reflected the mitral cells somatic responses, we analyzed the widefield responses as 2 Thy1-GCaMP6f WT mice that learned to associate target odors in our go/no-go paradigm as this condition matches the go/no-go behavior used in Lindeman et al. The WT mice were already trained to discriminate a first odor pair, that is to lick in response to isopropyl butyrate and to refrain from licking for isobutyl propionate both presented at a low concentration of 0.025% of saturated vapor pressure. On the first day, the animals were presented with a novel pair of target odors with propyl butyrate (S^+^) and ethyl propionate (S^-^) at 0.025% of saturated vapor pressure. The behavioral performance on the novel odor pair on the first day was close to chance levels (52.3%, 306 trials). The novel odor pair was presented again on a second day and the behavioral performance significantly improved (p = 0.0446, Fisher exact test) to 62.7% (150 trials). We recorded 62 glomeruli on the first day and 105 glomeruli on the second day. We quantified the odor response to the S^+^ for each ROI in each presentation as the average z-score over 1 second window starting at S^+^ onset, with responses with an average positive z-score considered excitatory. The ROIs had excitatory responses on most of the presentations of the S^+^ (go odor responses) with 72.5% being excitatory for the known S^+^ (2501 roi-presentations) on the first day. On the second day, excitatory responses still dominated with 78.8% being excitatory for the S + (2618 roi-presentations). Mitral cell somatic responses also show reduction in their responses to an odor with exposure (Kato et al. 2012). We have looked at the neural responses to the odors to test whether there was a significant reduction in response on the first day compared to the second day. The average response on the first day was 0.6176 ± 0.0242 z-score (mean ± s.e.m, n = 2749 responses). The average response did not become smaller (p = 0.9992, single tailed t-test) on the second day being 0.7205 ± 0.0218 (mean ± s.e.m, n = 2889 responses).The lack of inhibitory responses to the S^+^ and the lack of reduction of odor responses with exposure are not consistent with mitral cell somatic responses. **E.** Example of responses to go target odor in a WT mouse on the first day (naïve response) and the second day (expert response). Responses were dominated by excitatory (positive) responses. **F.** Average glomerular response (mean ± s.e.m) for naïve and expert mice (two mice). Download Figure 1-1, TIF file.

**Figure 2.**
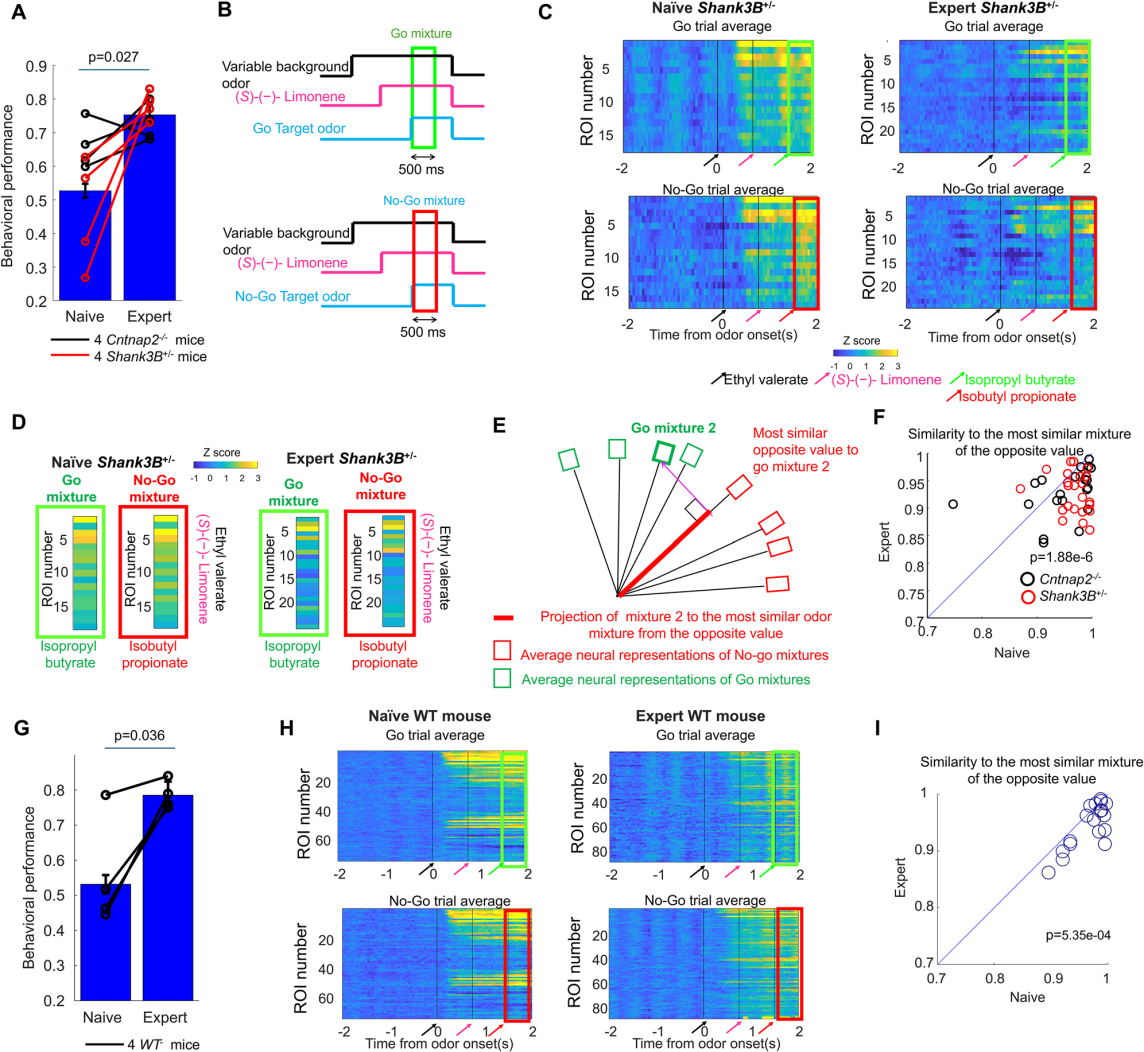
Behavioral training increased the discriminability of neural representations in the olfactory bulb in *Shank3B^−/+^* and *Cntnap2*^−/−^ as well as WT mice. Population neural responses were evaluated as mice transitioned from being exposed to the background odors for the first time at a concentration of 0.1% (naive condition) and when they achieved higher performance after training (expert condition). ***A***, Behavioral improvement was seen in four *Shank3B^−/+^* and four *Cntnap2*^−/−^ mice after training. Younger animals performed better in the naive condition but the effect of age on performance disappeared on the expert condition (Extended Data Fig. 2-1). ***B***, For each target and background mixture, we determined the population neural response as the average response on a 500 ms window starting at the onset of the target odor across responsive glomeruli. This window limited the effect on neural response produced by licking (Extended Data Fig. 2-2). ***C***, Examples of population responses in a *Shank3B^+/−^* mouse to mixtures of opposite value. ***D***, Averages taken over the 500 ms following target onset. The similarity between population responses was higher in the naive condition compared with the expert condition. ***E***, For each target and background mixture, we calculated the normalized dot product with the target and odor backgrounds of the opposite value to determine the similarity. We determined the closest match of the opposite value and used the similarity to best match of the opposite value to quantify the stimulus separability. ***F***, Closest match of the opposite value in the naive versus the expert condition for 64 target and background odor mixture from 4 *Cntnap2^−/−^* and 4 *Shan3B^+/−^* mice and 8 odor mixtures. For most of the mixtures, there was a decrease in similarity to the closest match of the opposite value in the expert condition compared with the naive condition. ***G***, Four WT also show improvement in target recognition with background with training. ***H***, Example of population responses in a WT mouse showing an improvement in discriminability of mixtures of opposite value. ***I***, For most of the odor mixtures in WT mice (32 target and background mixtures), there was also an improvement in stimulus discriminability as quantified using the similarity to best match of the opposite value.

10.1523/ENEURO.0271-25.2025.f1-2 Figure 1-2 **Age and sex of animals used** Download Figure 1-2, TIF file.

We used wide-field calcium imaging in head-fixed, behaving *Cntnap2*^−/−^ (Poliak et al., 2003) and *Shank3B^−/+^* (Peça et al., 2011) mice crossed to GCaMP6f reporter mice (C57BL/6J-Tg(Thy1-GCaMP6f) GP5.11Dkim/J). The GP5.11 reporter line expresses GCaMP6f in the mitral and tufted cells of the olfactory bulb (Dana et al., 2014) and has been used to monitor the output of the olfactory bulb using wide-field imaging (Storace and Cohen, 2017). All ASD mouse crosses showed similar GCaMP6f expression as in WT GCaMP6f mice (Fig. 1*C*). Labeled glomeruli were visible in histology sections and robust odor-evoked responses from individual glomeruli were evident after applying a high-pass spatial filter (see Fig. 1*D*,*E* and see attached videos for examples of odor responses. Movie 1 is from a WT mouse, Movie 2 is from a *Shank3B^−/+^* mouse, and Movie 3 is an example from a *Cntnap2*^−/−^ mouse). ROIs were drawn over responsive glomeruli. Glomerular responses were quantified as *z*-scores using the mean and standard deviation of the air period as previously described (Li et al., 2023).

**Movie 1.** Example of average glomerular activity (10 repeats) of a WT mouse in response to ethyl butyrate and (s)-(−)limonene at 0.1% of saturated vapor pressure as backgrounds, with the no-go target odor being ethyl propionate at 0.025% of saturated vapor pressure. Images are averaged over 750 ms time windows. Glomerular responses were seen in all 3 genotypes (see attached videos). [View online]

**Movie 2.** Example of average glomerular activity (10 repeats) of a *Shank3B^+/−^* mouse in response to ethyl butyrate and (s)-(−)limonene at 0.1% of saturated vapor pressure as backgrounds, with the no-go target odor being ethyl propionate at 0.025% of saturated vapor pressure. Images are averaged over 750 ms time windows. Glomerular responses were seen in all three genotypes (see attached videos). [View online]

**Movie 3.** Example of average glomerular activity (10 repeats) of a *Cntnap2^−/−^* mouse in response to ethyl butyrate and (s)-(−)limonene at 0.1% of saturated vapor pressure as backgrounds, with the no-go target odor being ethyl propionate at 0.025% of saturated vapor pressure. Images are averaged over 750 ms time windows. Glomerular responses were seen in all three genotypes (see attached videos). [View online]

Although the GP5.11 reporter line expresses GCaMP6f in mitral and tufted cells in the olfactory bulb, it also expresses GCaMP6f in areas that send axons into the olfactory bulb, including the piriform cortex, hippocampus, and entorhinal cortex (Dana et al., 2014). The raw fluorescence signal recorded from the olfactory bulb includes activity from olfactory bulb neurons as well as bulbar projecting axons from these other parts of the brain. However, the glomerular pattern that we observe with our spatially high-pass filtered fluorescent signal mainly represents activation of neurons in the olfactory bulb, as odor responses from individual glomeruli were not apparent in a *Shank3B^−/+^* mouse that was injected with *GCamp6F* virus in the piriform cortex, despite the massive axonal projection from the piriform cortex into the olfactory bulb (Extended Data Fig. 1-1*A–D*). The feedback axon inputs are diffusely distributed at the spatial scale of individual glomeruli (Boyd et al., 2015) and were substantially reduced by the high-pass spatial filtering used. Previous work has shown that mitral cell somatic responses were suppressed by reward association in WT mice (Lindeman et al., 2024). Using our wide-field approach, we did not observe suppressed responses in WT mice with reward association (Extended Data Fig. 1-1*E,F*). Our data is more consistent with reflecting data from mitral and tufted cells dendrites.

Mice (4–10 months old; see Extended Data Fig. 1-2 for the age and sexes of the mice used) were trained to identify target odors in the presence of background odors (see Fig. 1*B* and see Li et al., 2023 for detailed training protocol). Briefly, water-deprived mice were first trained on a simple odor discrimination task which required licking a water tube for the go target odors and to refrain from licking for the no-go target odors. For each trial, a single target odor was presented. There were two possible go target odors and two no-go target odors. Correct licking was immediately rewarded with 4 µl of water (hits). Incorrect licking (false alarms) resulted in time-out. After mastering the pure targets task at a low concentration of 0.02% of saturated vapor pressure (∼5 d of training), the mice were introduced to background odors that started before the onset of the target odors and later overlapped with the targets odors. The background odor made the go/no-go odors more difficult to discriminate (Li et al., 2023). The early onset of the background odors respect to the targets allowed for comparison of the neural representation of background odors alone without the targets. Each trial contained two different background odors. The first was a variable background odor which was chosen from one out of four possible odors. These four variable background odors were used across all trials and increased the overall similarity of the glomerular representation between the go stimulus and the no-go stimulus (Li et al., 2023). After 750 ms from the onset of the first background odor, the second background odor, (s)-(−)-limonene, appeared. After 750 ms from the onset of (s)-(−)-limonene, the target odor appeared overlapping with the first and second background odors. The mixture of target and backgrounds was continuously delivered for 3 s. In case the animal licked, the odor delivered was stopped after 1 s from the first lick. The background odor concentrations were increased gradually over 3 d to a final high concentration of 0.1% of saturated vapor pressure in order to make the discrimination of target odors more challenging. The weak target odors (0.025% vapor saturation) activated a smaller fraction of glomeruli (29.5% ± 2.0%, mean ± SD) compared with the stronger (0.1% vapor saturation) background odors (43.5% ± 6.2%, mean ± SD) as previously determined using intrinsic imaging. The glomeruli activated by the background odors overlapped with the target odors, resulting in effective masking and increasing the difficulty of the task (Li et al., 2023).

### Behavioral training increased neural discriminability of odor mixtures in Shank3B^−/+^ and Cntnap2^−/−^ mice

Both *Shank3B^−/+^* and *Cntnap2^−/−^* mice readily learned to discriminate go odors from no-go odors but performance dropped when background odors are introduced. During initial training sessions (Fig. 2*A*,*B*) when mice are “naive” to the background odors, behavioral performance was low: *Shank3B^−/+^* mice performed at 45.9 ± 16.6% (mean ± SD, 1,254 trials) and *Cntnap2^−/−^* mice at 65.9 ± 7.0% (1,049 trials). After an average of 3.3 ± 2.4 training sessions, both mouse models became “experts,” where performance significantly improved (paired *t* test, *p* = 0.027, *n* = 8 mice). In the expert condition, *Shank3B^−/+^* mice reached 78.1 ± 4.2% (373 trials) and *Cntnap2^−/−^* mice 72.8 ± 5.5% (400 trials) accuracy. Training improves performance on target discrimination in the presence of background odors. There was higher variability in behavioral performance in the naive condition across mice (Extended Data Fig. 2-1). This variability was partially explained by age, with younger mice performing better. Specifically, performance in the naive condition was negatively correlated with age (*r* = −0.72, *p* = 0.009, *n* = 12 mice). However, this effect of age was no longer present in expert condition, where the correlation between age and performance was not significant (*p* = 0.12).

10.1523/ENEURO.0271-25.2025.f2-1 Figure 2-1 **Behavioral performance and age.** Younger mice performed better in the naïve condition compared to older mice. The difference disappeared in the expert condition. Each symbol is an individual mouse. Download Figure 2-1, TIF file.

10.1523/ENEURO.0271-25.2025.f2-2 Figure 2-2 **Glomerular responses precede the licking action.** Average glomerular responses for *Shank3B^+/-^* and *Cntnap2^-/-^* mice for hits with background odors aligned to the first lick, as measured as the electrical contact with the licking tube. An average licking response was calculated for each session (12 sessions for the *Shank3B^+/-^* mice and nine sessions for the *Cntnap2^-/-^* mice). Lines are mean ± s.e.m. There was an elevation in the neural response >200 ms that preceded the licking response. Download Figure 2-2, TIF file.

Based on these behavioral findings, we hypothesized that training to detect target odors within background odors would enhance the neural discriminability of target–background odor mixtures. To test this, we used wide-field calcium imaging to compare olfactory bulb responses in four *Shank3B^−/+^* and four *Cntnap2^−/−^* mice under two conditions: before (naive) and after (expert) training with the background odors. In both conditions, we presented the same set of eight odor mixtures, composed of four “go” mixtures (containing rewarded target odors at 0.025% of saturated vapor pressure) and four “no-go” mixtures (containing unrewarded target odors at 0.025% vapor pressure), all paired with background odors at a higher concentration of 0.1% of saturated vapor pressure.

To quantify neural discriminability, we compared the population neural representations evoked by go versus no-go mixtures in both naive and expert conditions. Because licking behavior induces neural activity beginning ∼200 ms before the response (Extended Data Fig. 2-2), we designed our analysis to minimize this confound. First, we included only correct trials (hits and correct rejections) to control for performance differences between naive and expert animals. Second (Extended Data Fig. 2*B*), we restricted our analysis to a 500 ms window following target odor onset—well before the average lick times (*Cntnap2^−/−^*: 1,185.5 ± 671 ms; *Shank3B^−/+^*: 1,172 ± 588 ms; mean ± SD)—to ensure that responses reflected odor processing rather than motor output.

We generated population response vectors for each odor mixture by averaging glomerular activity across this 500 ms window (Fig. 2*C*,*D*). A glomerulus was included in the analysis if it responded to any of the eight mixtures with an average activity >1 *z*-score in either the naive or expert condition. The resulting vectors were normalized to unit length. For each go target mixture, we identified the most similar no-go mixture (and vice versa) based on the dot product between their population vectors. The higher the dot product, the greater the similarity between the neural representations (Fig. 2*E*). This approach allowed us to assess how well go and no-go mixtures were separated in the neural space and how this changed with training.

For most odor mixtures, training led to a decrease in similarity to the most similar opposite-value mixture (*p* = 1.88 × 10^−6^, sign test, 51/64 animal–odor pairs across 4 *Cntnap2^−/−^* and 4 *Shan3B^+/−^* mice; Fig. 2*F*). Thus, behavioral improvement was accompanied by an increase in the average neural discriminability between odor mixtures.

### Behavioral training increased the discriminability of average neural representations with background odors in WT mice

To determine whether the increase in neural discriminability also occurred in WT mice, we also trained and tested four C57BL/6J-Tg(Thy1-GCaMP6f) GP5.11Dkim/J (WT) mice with the same paradigm as the *Shank3B^+/−^* and *Cntnap2^−/−^* mice (Fig. 2*G*). The performance with background odors in the naive condition was impaired (55.3 ± 15.8%, *n* = 4 *WT* mice, mean ± SD, 1,416 trials). However, after 3 ± 2.3 sessions of practice, the performance also significantly improved (paired *t* test, *p* = 0.036, *n* = 4 mice) to expert levels of accuracy 78.5.0 ± 4.0% (mean ± SD, 400 trials). The increase in performance with training (+23.2 ± 12.7%, *n* = 4 WT mice) was not significantly different (*p* = 0.75, double-tailed *t* test) from the increase in performance with training of the *Shank3B^+/−^* and *Cntnap2^−/−^* mice (+19.5± ± 19.9%, *n* = 8 mice). For most of the odor mixtures, there was also a decrease in the similarity of the most similar opposite-value mixture with training (*p* = 5.35 × 10^−4^, sign test, 26/32 animal–odor pairs from 4 *WT* mice; Fig. 2*H*,*I*). Thus, behavioral improvement was associated with an increase in the average neural discriminability between odor mixtures in both mouse models of autism and WT mice.

### Behavioral training reduced the variability of neural representations in the olfactory bulb in *Shank3B^−/+^* and *Cntnap2^−/−^* mice

**Figure 3.**
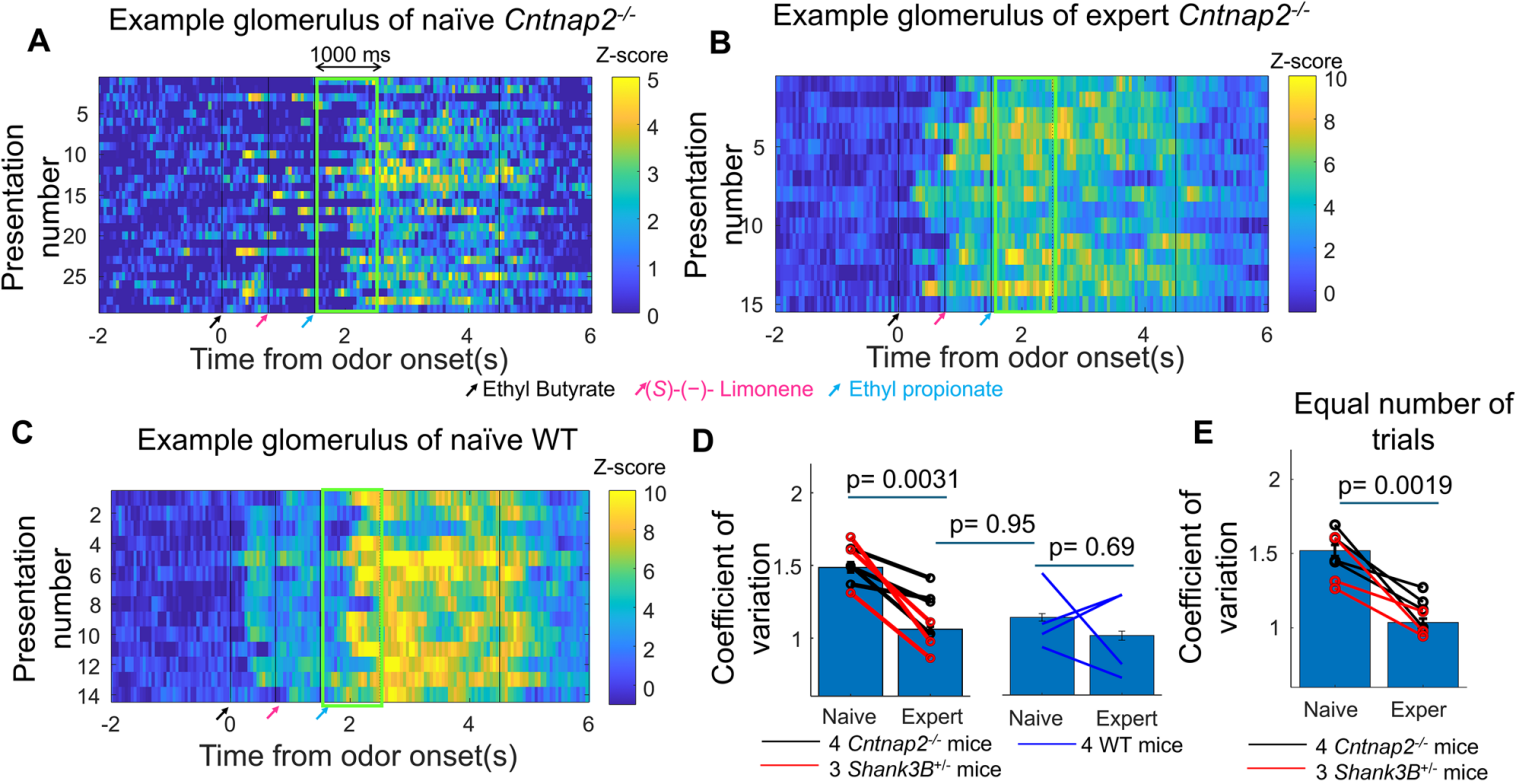
Behavioral training reduced the variability of neural representations in the olfactory bulb in *Shank3B^−/+^* and *Cntnap2*^−/−^ mice. ***A***, Example of the response of a glomerulus of a *Cntnap2*^−/−^ mouse to a no-go mixture in the naive condition, showing variable responses to different presentations of the mixture. ***B***, Response of a glomerulus in the expert condition of the same *Cntnap2*^−/−^ mouse, showing less trial-by-trial variability. ***C***, Example of a stable response of a glomerulus in a naive WT mice. ***D***, Neural responses per glomeruli per trial were quantified as the average response in the 1 s following the target onset for correct rejections. The coefficient of variation was calculated for each ROI–odor per mice and is shown in the bar graphs. The average coefficient of variation per mice is shown in individual symbols was reduced in *Shank3B^−/+^* and *Cntnap2*^−/−^ mice in the expert condition. Coefficient of variation in naive WT mice was similar to the already trained mouse models of ASD. ***E***, Coefficient of variation calculated using the same number of trials between naive and expert mouse models of ASD show similar increase in reliability in the expert condition.

Stimulus discrimination does not only depend on the average neural responses, but also on the neural variability, with increased neural variability associated with poor discrimination (Faisal et al., 2008). Increased variability has been observed in other sensory modalities in mouse models of autism (Bhaskaran et al., 2023). We wondered whether increased practice with a novel background odor would reduce the neural variability during the target presentation, potentially contributing to the improved behavioral discrimination in *Shank3B^−/+^* and *Cntnap2^−/−^* mice as they transitioned from being naive to being experts. To avoid potential variability in odor responses produced by differences in licking response, we only analyzed correct rejections, where there is no licking. To have robust statistics of the trial-by-trial variability, we analyzed the responses where the mice performed at least 20 trials of correct rejections for both the naive and expert condition and the glomerular responses were evaluated in a longer window of 1 s following the onset of the target odor (Fig. 3).

For each individual glomerulus–odor mixture, we calculated a standard deviation and a mean for all the presentations and calculated the coefficient of variation as the standard deviation divided by the mean. For a glomerulus to be included in the analysis, we required it to have at least one response to a no-go stimulus of *z*-score >1. For a glomerulus–odor mixture to be included in the variability analysis, we required that there were at least six repeats with an average *z*-score of 0.1 or larger. We analyzed the data from 4 *Cntnap2^−/−^* mice and 3 *Shank3B^+/−^* mice, as one of the *Shank3B^+/−^* mouse did not have enough trials to assess variability.

The variability was indeed higher for the naive condition with a coefficient of variation of 1.49 ± 0.03 (mean ± SEM, *n* = 1,312 glomerulus–odor pairs) and odor responses became more reliable in the expert condition, with a coefficient of variation of 1.06 ± 0.03 (mean ± SEM, *n* = 1,323 glomerulus–odor pairs). The average coefficient of variation per mixture of background and target odor per glomerulus decreased for all animals tested with behavior (*p* = 0.0031, double-tailed *t* test, *n* = 4 *Cntnap2^−/−^* mice and 3 *Shank3B^+/−^* mice) as *Cntnap2^−/−^* and *Shank3B^+/−^* mice transitioned from being naive to being experts, potentially contributing to the improved performance with background odors with experience.

Differences in the number of repeats between the naive and expert conditions could potentially bias the estimation of variability. To test whether our conclusions were robust to this factor, we determined, for each animal and each odor mixture, the number of trials available in the naive and expert conditions. We then recalculated the coefficient of variation using the same number of trials in both conditions by randomly subsampling trials from the condition with more repeats to match the other. Even after this control, variability remained higher in the naive condition (coefficient of variation = 1.52 ± 0.04, mean ± SEM, *n* = 1,099 glomerulus–odor pairs) compared with the expert condition (1.04 ± 0.03, mean ± SEM, *n* = 1,108 glomerulus–odor pairs, *p* = 0.0019, double-tailed *t* test, *n* = 4 *Cntnap2^−/−^* mice and 3 *Shank3B^+/−^* mice; Fig. 3*E*).

### Low variability of neural representations in naive WT mice

Naive *Shank3B^−/+^* and *Cntnap2^−/−^* mice target odor responses became more stable after training. We wondered whether this stabilization of odors responses was a particular feature of mouse models of ASD or whether the WT mice also required training to stabilize their neural responses.

The target odor responses in naive WT mice (*n* = 4) were as reliable as the *Shank3B^−/+^* and *Cntnap2^−/−^* mice following training (Fig. 3*C*,*D*). Naive WT mice CV was 1.14 ± 0.03 (4 WT mice, mean ± SEM, *n* = 1,082 glomerulus–odor pairs) similar to the CV of the expert *Shank3B^+/−^* and *Cntnap2^−/−^* mice (1.06 ± 0.03, mean ± SEM, *n* = 1,323 glomerulus–odor pairs). The differences of the average CV per animal was not significantly different (*p* = 0.95, double-tailed *t* test) between naive WT mice (1.12 ± 0.22, mean ± SD, *n* = 4 WT mice) and the expert mouse models of ASD (1.13 ± 0.19, mean ± SD, *n* = 7 mice, 4 *Cntnap2^−/−^* and 3 *Shank3B^−/+^* mice). In the WT mice, there was no significant increase (*p* = 0.69, double-tailed paired *t* test) in average reliability with training with a CV coefficient of variation of 1.01 ± 0.03 after training (mean ± SEM, *n* = 1,133 glomerulus–odor pairs).

Thus, naive WT mice responses were more reliable than naive mouse models of ASD and experience did not improve the trial-by-trial variability. In contrast, training was necessary for the mouse models of ASD to stabilize their responses.

### Fluctuations in earlier background odors responses affected later responses during the target odor presentation in naive *Shank3B^−/+^* and *Cntnap2^−/−^* mice

Neural responses to individual presentations of target and background odors exhibited substantial trial-to-trial variability in naive *Shank3B^+/−^* and *Cntnap2*^−/−^ mice. Early activation of mitral cells in the olfactory bulb can cause prolonged depolarizations that influence later responses (Matsumoto et al., 2009). We wondered whether large fluctuations in neural activity during the background odor period—prior to target onset—would be followed by similarly large fluctuations during the subsequent target and background presentation period, potentially impairing target discrimination.

**Figure 4.**
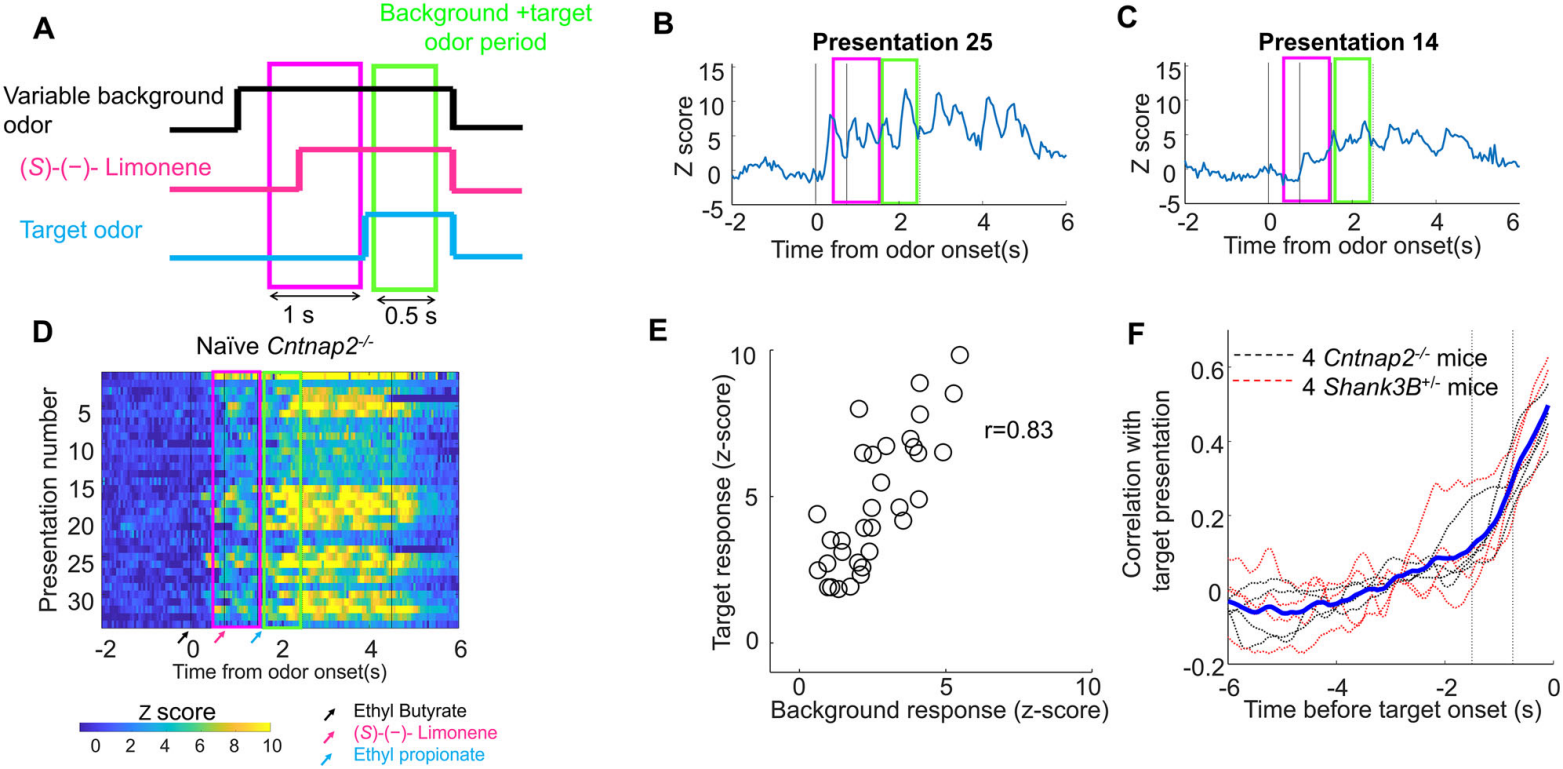
Fluctuations in background odors responses affected responses during the target odor presentation in naive *Shank3B^−/+^* and *Cntnap2*^−/−^ mice r. ***A***, Responses to the same odor mixtures were analyzed on individual presentations. ***B***, Example of a single glomerulus responses in a *Cntnap2*^−/−^ mouse response showing a trial where the background odor produced a large response, and there was also a large response during the target and background period. ***C***, Trial where the same background (as in ***B***) produced a small response and there was also a small response during the target and background period. ***D***, Example showing responses of a single glomerulus to multiple repeats of the same backgrounds and target combination. ***E***, Example of the strong linear correlation between the response in the 1 s period preceding the onset of the target and the 1 s after the target onset for a single ROI in a *Cntnap2*^−/−^ mouse. Individual circles represent individual trials. ***F***, There was strong linear correlation between the 1 s response to the target and background mixture and the background responses preceding the target for *Shank3B^−/+^* and *Cntnap2*^−/−^ mice. Background responses were evaluated in a 1 s sliding window. The blue line is the average correlation across all animals.

We analyzed how the trial-by-trial variability in individual glomerular responses during the background odor period influenced the neural response during the subsequent target odor period in naive *Shank3B^−/+^* and *Cntnap2*^−/−^ mice (Fig. 4*A–C*). We examined each of the eight possible background–target odor combinations separately. Neural responses to both background and target odors varied markedly across trials. Notably, trials in which the background odor alone evoked strong neural responses were also trials in which the combined background and target odors elicited stronger responses, suggesting a modulatory effect of the background-evoked activity on the subsequent target representation (see Fig. 4*D* for an example of a single glomerulus response to multiple presentations of the same odor mixture).

We calculated the correlation coefficient on individual glomeruli of the neural response between the background odor period and the background and target odor period (Fig. 4*E*). We quantified the target and background mixture response in the 1 s window after the target onset. We calculated the correlation with a similar duration a 1 s sliding window, for each odor presentation (Fig. 4*F*). During the preceding second, the average correlation was large for the *Cntnap2^−/−^* mice (0.46 ± 0.08, mean ± SD, *n* = 4 mice) as well as for the *Shank3B^+/−^* mice (0.53 ± 0.09, mean ± SD, *n* = 4 mice). This correlation was mostly related to the odor-evoked activity. The correlation in the air period just preceding the background onset was lower for the *Cntnap2^−/−^* (0.12 ± 0.08) and *Shank3B^+/−^* mice (0.11 ± 0.13), and it was significantly lower than the correlation with the background period (*p* = 7.98 × 10^−5^, double-tailed paired *t* test). The activity produced during the target odor presentation in *Shank3B^+/−^* and *Cntnap2^−/−^* mice on individual glomeruli was affected by activity on the 2 s preceding the onset of the target. As there was no information regarding the target identity during the background period, these fluctuations could potentially be detrimental for determining the identity of the target odors.

### Reduced responses to background odors increased the discriminability of neural representations in the olfactory bulb in naive *Shank3B^−/+^* and *Cntnap2^−/−^* mice

**Figure 5.**
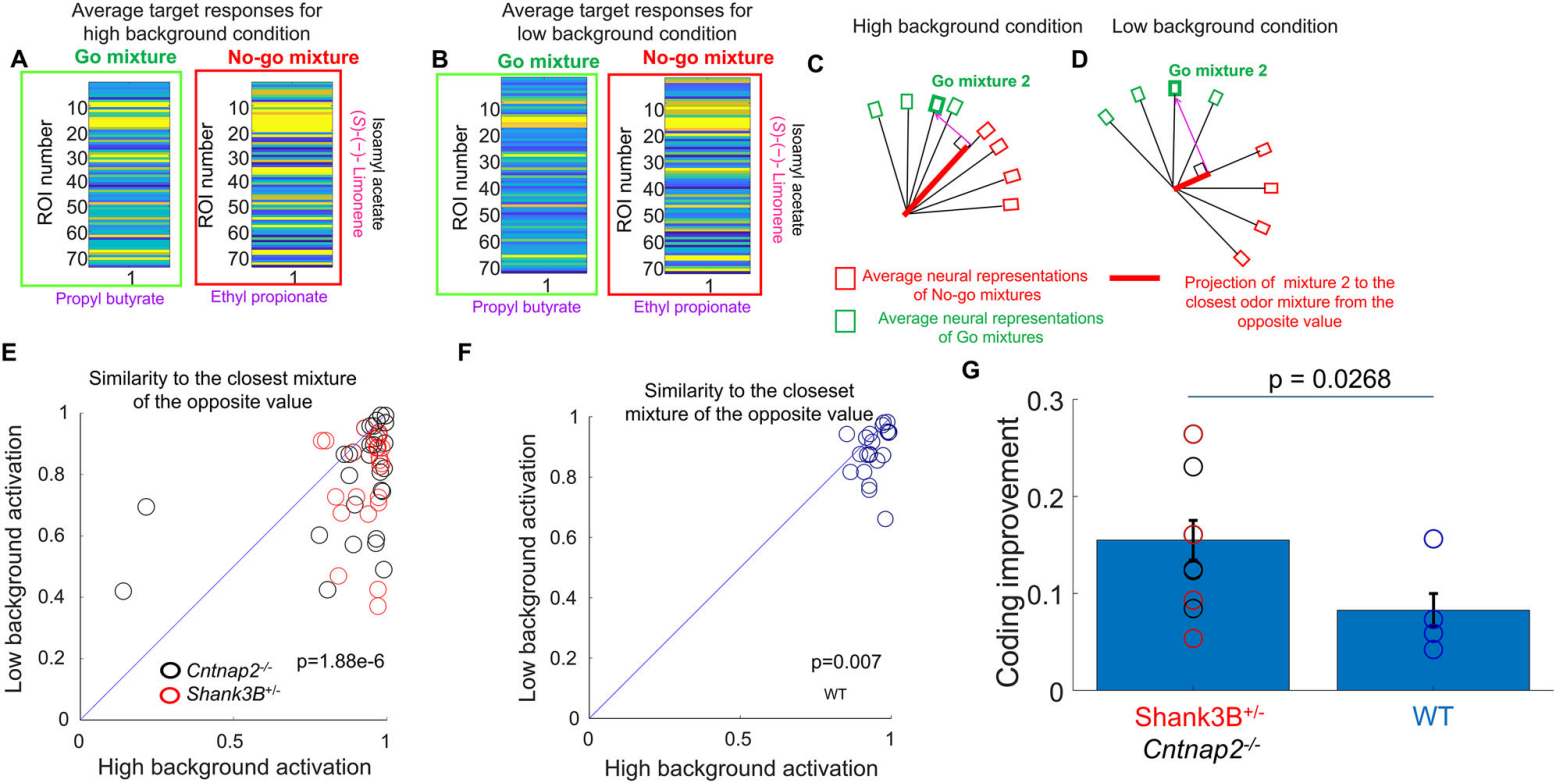
Fluctuations in background odors in naive *Shank3B^−/+^* and *Cntnap2*^−/−^ mice reduced the discriminability of neural representations. ***A***, ***B***, We separated trials in the naive condition based on the background odor response as high background or low background. Example of population neural responses in a *Shank3B^−/+^* mouse to the same mixture for a high background and a low background response, where each odor matched with closest match of the opposite value. Population neural responses were larger and more similar to its closest match of the opposite value in the high background condition compared with the low background condition. ***C***, ***D***, For each target and background mixture, we calculated the normalized dot product with the target and odor backgrounds of the opposite value and determined the closest match of the opposite value for both the high background condition and the low background condition. ***E***, Closest match of the opposite value in the high background versus the low background condition for 64 target and background odor mixture from 4 *Cntnap2^−/−^* and 4 *Shan3B^+/−^* mice and 8 odor mixtures. For most of the mixtures, there was a decrease in similarity to the closest match of the opposite value in the low background condition compared with the high background condition. The decrease in similarity in the low background condition did not depend on the vapor pressure of the target or the background odors (Extended Data Fig. 5-1). ***F***, Closest match of the opposite value in the high background versus the low background condition for 32 target and background odor mixture from 4 WT mice. There was also a decrease in similarity to the closest match of the opposite value in the low background condition compared with the high background condition. ***G***, Coding improvement was defined as the reduction in similarity to the nearest neighbor of the opposite value seen with low background activation. This coding improvement was larger for mouse models of ASD. Symbols represent individual mice coding improvement averaged over all odor mixtures.

In naive *Shank3B^−/+^* and *Cntnap2*^−/−^ mice, trials with elevated neural responses during the background odor period were associated with increased activity during the subsequent target and background odor presentation. To assess whether this elevated background activity impacted odor discrimination, we categorized trials based on the magnitude of background responses into high and low response groups. Trials were classified as low background response if the glomerular activity in the 1 s prior to target onset was in the lowest 25th percentile for a given background odor and as high response if it was in the highest 75th percentile. This classification was done separately for each background odor to isolate the effect of trial-by-trial fluctuations in response magnitude from differences in odor identity (Fig. 5*A–D*).

10.1523/ENEURO.0271-25.2025.f5-1 Figure 5-1 Coding improvement produced by lower background activation did not depend on the background nor target odor vapor pressure in *Cntnap2^-/-^* and *Shank3B^+/-^* mice. Coding improvement was defined as the reduction in similarity to the nearest neighbor of the opposite value seen with low background activation. Each error bar is the mean ± s.em of average coding improvement for the background odors and target odors for all 4 *Cntnap2^-/-^* and 4 *Shank3B^+/-^* mice. There was no monotonic relationship between the vapor pressure and the coding improvement produced in trials with reduced background activation. Download Figure 5-1, TIF file.

We then compared neural discriminability across these trial types by calculating the similarity between population vectors evoked by target–background mixtures and their most similar opposite-value mixtures. For each of the eight target–background combinations, we constructed glomerular population vectors using activity in the 500 ms following target onset, including only glomeruli with at least one average response exceeding 1 *z*-score. Analyses were restricted to correct trials to control for potential behavioral confounds such as licking. Across animals and mixtures (Fig. 5*E*), we found that high background response trials were associated with significantly greater similarity to opposite-value mixtures (*p* = 1.88 × 10^−6^, sign test, 51 out of 64 odor–animal pairs), indicating reduced neural discriminability.

In naive *Shank3B^−/+^* and *Cntnap2*^−/−^ mice, spontaneous fluctuations in background odor responses influenced the fidelity of target representation. The four background odors used in this study had vapor pressures ranging from 4.8 mmHg (ethyl valerate) to 12.8 mmHg (ethyl butyrate). We asked whether the improved neural discriminability observed in trials with lower background activation was specific to low vapor pressure odors. To quantify this, we measured coding improvement as the change in similarity to the nearest opposite-value mixture between low and high background response trials. Across animals and odors, coding improvement was not correlated with the vapor pressure of the background odors (*R* = −0.04, *p* = 0.77; Extended Data Fig. 5-1) or of the target odors (*R* = −0.01, *p* = 0.95).

Trials with lower background activation produced more distinct neural representations of target–background mixtures, whereas elevated background responses were associated with reduced discriminability between go and no-go stimuli.

### Reduced background responses improve neural discriminability in naive WT mice, but less than in ASD models

To determine whether reduced responses to background odors increased the discriminability of neural representations in the olfactory bulb in WT mice, we applied the same analysis to naive WT animals. Reduced background odor responses were also associated with improved neural discriminability of target–background mixtures, although the effect was less pronounced than in *Shank3B^−/+^* and *Cntnap2*^−/−^ mice. As with the ASD models, we compared trials with low and high background activation based on the glomerular responses preceding target onset. In low background response trials, the neural representation of a given mixture was more distinct from its most similar opposite-value counterpart. Across odor pairs per animal (Fig. 5*F*), we observed a significant reduction in similarity to the most similar opposite-value mixture in low background trials (*p* = 0.007, sign test, 24 out of 32 pairs from 4 WT mice). To quantify this effect, we used the coding improvement as defined above. In WT mice (Fig. 5*G*), the average coding improvement was 0.082 ± 0.017 (mean ± SEM, 24 odor pairs), significantly smaller than the improvement observed in *Shank3B^+/−^* and *Cntnap2^−/−^* mice (0.155  ±  0.020, mean ± SEM, 51 pairs; *p* = 0.027, two-tailed *t* test). These results suggest that while fluctuations in background activity can impact neural discriminability in WT mice, their influence is more limited compared with the heightened sensitivity observed in ASD models.

### Background odor responses were reduced as *Shank3B^+/−^* and *Cntnap2^−/−^* mice transition from being naive to being experts

In naive *Shank3B^+/−^* and *Cntnap2^−/−^* mice, neural representations of odor mixtures containing target go and no-go components were more similar than in the expert condition, where these representations became more distinct. However, even in the naive state, some trials exhibited reduced neural activation during the early background odor period, and these trials were associated with more distinct representations of the subsequent mixtures. This observation suggests that, with experience, reduced early responses to background odors may contribute to enhanced neural discriminability of odor mixtures in *Shank3B^+/−^* and *Cntnap2^−/−^* mice.

**Figure 6.**
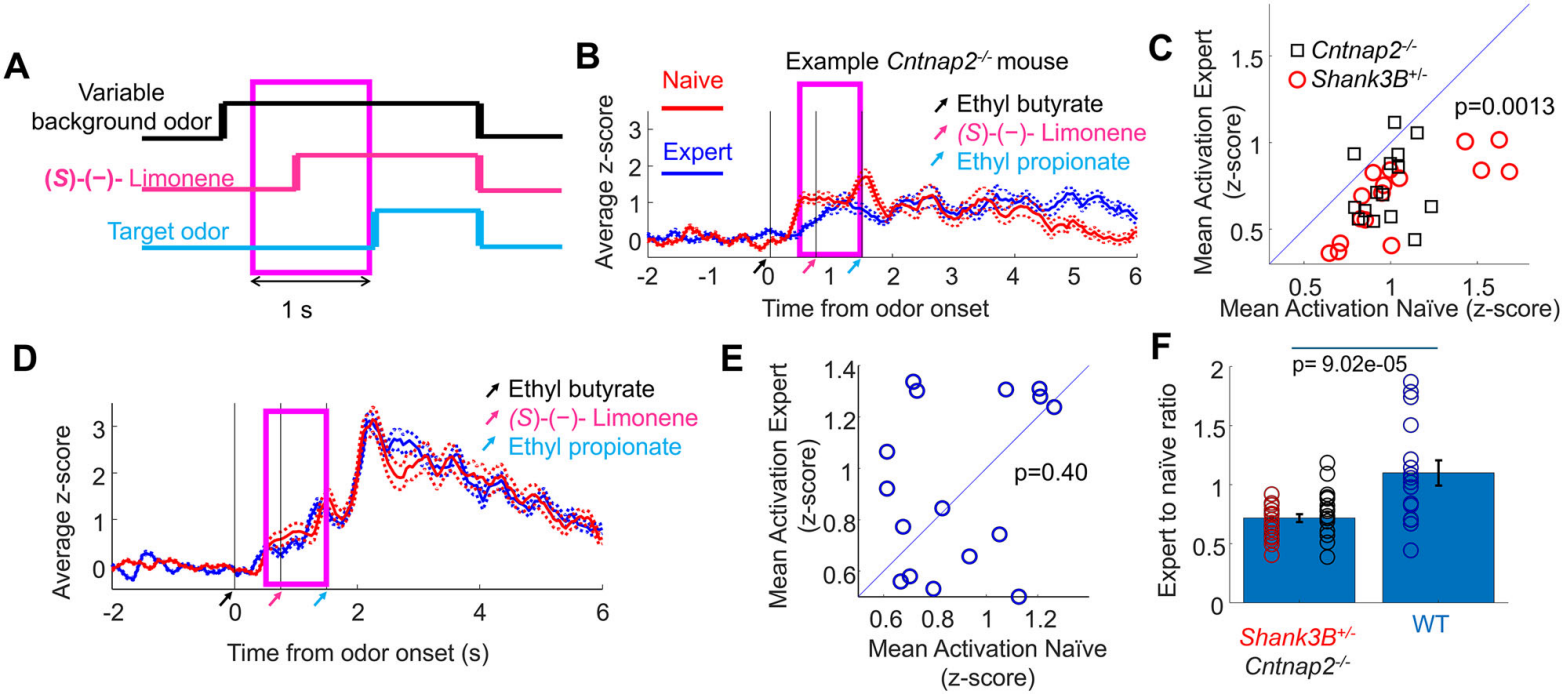
Background odor responses decrease as *Shank3B^+/−^* and *Cntnap2^−/−^* mice transition from being naive to being experts. ***A***, Background responses were quantified in a 1 s window that preceded the onset of the target onset. ***B***, Example of an average glomerular response (mean ± SEM) in a *Cntnap2^−/−^* mice to an odor mixture, in the naive condition and the expert condition. The average glomerular response during the background presentation was reduced in the expert condition. ***C***, Mean average glomerular responses for background odors were reduced with experience for most of the animal–odor pairs in *Shank3B^+/−^* and *Cntnap2^−/−^* mice. Target odor responses were not reduced as *Shank3B^+/−^* and *Cntnap2^−/−^* mice transition from being naive to being experts (Extended Data Fig. 6-1). ***D***, Example of the average glomerular response in a WT mouse to an odor mixture in the naive condition and the expert condition. The average glomerular response during the background presentation was not reduced in the expert condition. Sniffing responses to background odors did not change with experience in *Shank3B^+/−^* and *Cntnap2^−/−^* mice (Extended Data Fig. 6-2). ***E***, Mean average glomerular responses for background odors were not broadly reduced with experience in WT mice. Sniffing responses to background odors did not change with experience in WT mice (Extended Data Fig. 6-3). ***F***, The reduction in background responses with training was larger in mouse models of ASD compared with WT mice.

The early neural response to the background odors was indeed reduced as *Shank3B^+/−^* and *Cntnap2^−/−^* mice behavior transitioned from being naive to being experts (Fig. 6*A*,*B*). We quantified the neural response to the background as the average neural response during the 1 s preceding the onset of the target onset. We included glomeruli that responded during the target and background odors period with at least *z*_score > 1 during the 500 ms after target onset for any of the eight used target and background combinations in either the naive or the expert condition. The response to the background in the naive mice (Fig. 6*C*) was 1.04 ± 0.02 *z*-score (mean ± SEM, 2,505 ROI–odor responses), and it was significantly larger [*p* = 0.0013, double-tailed *t* test, with *n* = 8 mice (4 *Cntnap2^−/−^* mice and 4 *Shank3B^+/−^* mice) than the background responses in the *Shank3B^+/−^* and *Cntnap2^−/−^* mice after they became experts (0.70 ± 0.01 *z*-score, mean ± SEM, 2,148 ROI–odor pairs]. We also measured the mean glomerular activation for individual background odor mixtures in each animal. Out of the 31 background odor mixtures measured in 4 *Cntnap2^−/−^* mice and 4 *Shank3B^+/−^* mice in the naive and expert condition, 29 odor mixtures showed suppression in their mean glomerular activation with experience (*p* = 2.31 × 10^−7^, binomial test).

10.1523/ENEURO.0271-25.2025.f6-1 Figure 6-1 Target odor responses were not reduced as *Shank3B^+/-^* and *Cntnap2^-/-^* mice transition from being naïve to being experts. A. Target responses were quantified in a 500 ms window following the target onset. **B.** Mean average glomerular responses for target and background odors were not reduced with experience in *Shank3B^+/-^* and *Cntnap2^-/-^* mice. Download Figure 6-1, TIF file.

10.1523/ENEURO.0271-25.2025.f6-2 Figure 6-2 **Sniffing responses to background odors did not change with experience in *Shank3B^+/-^* and *Cntnap2^-/-^* mice. A.** Sniff responses to the backgrounds were evaluated in a 1 s window preceding the onset of the target odor. The target and odor mixtures were evaluated in a 1 s window following the onset of target odor. **B.** There was not significant difference in the sniff rate during the background period in between naïve and expert condition in the *Shank3B^+/-^* (in red) and *Cntnap2^-/-^* mice (in black). Lines represent individual animals. There was a significant increase in sniffing during the target period for the expert condition. **C.** The variability in the sniffing was unchanged between the naïve and the expert condition for *Shank3B^+/-^* and *Cntnap2^-/-^* mice. Download Figure 6-2, TIF file.

10.1523/ENEURO.0271-25.2025.f6-3 Figure 6-3 **Sniffing responses to background odors did not change with experience in WT mice. A.** Sniff responses to the backgrounds were evaluated in a 1 s window preceding the onset of the target odor. The target and odor mixtures were evaluated in a 1 s window following the onset of target odor. **B.** There was not significant difference in the sniff rate during the background period in between naïve and expert condition in the WT mice (in black). Lines represent individual animals. There was also no significant increase in sniffing during the target and odor period for the expert condition. **C.** The variability in the sniffing was unchanged between the naïve and the expert condition for WT mice. D-E. Naïve WT mice increased their sniff rate respect to the baseline for the background and target periods similar to expert mouse models of ASD. WT mice kept the increase in sniffing as experts. Download Figure 6-3, TIF file.

Was the suppression of early background response part of a general suppression of all odor responses? General odor response suppression could have resulted in suppression of the target and background presentation period, which might be detrimental to target discrimination. However, suppression of background odors in the expert *Shank3B^+/−^* and *Cntnap2^−/−^* mice did not extend to the target and background presentation period (Extended Data Fig. 6-1). We analyzed responses to correct no-go responses, to avoid potential influences of licking action. The mean glomerular response to the background and target period during the 1 s following the target onset in the naive mice was 1.47 ± 0.02 (mean ± SEM, 1,444 ROI–odor responses), and it was not significantly larger (*p* = 0.92, single tailed *t* test, from 4 *Cntnap2^−/−^* mice and 4 *Shank3B^+/−^* mice) than the background and target responses in the animals after they became experts (1.91 ± 0.03, mean ± SEM, 1,392 ROI–odor pairs). The suppression of background responses was specific to the moment before the target onset. Thus, behavioral training reduced the early responses to the background in the *Shank3B^+/−^* and *Cntnap2^−/−^* mice, constituting a mechanism for increasing the neural discriminability of embedded targets within background odors in these two mouse models of autism.

### Reduced background odor responses in expert *Shank3B^+/−^* and *Cntnap2^−/−^* mice were not caused by changes in sniffing rate

We wondered whether the reduction in olfactory bulb responses during background presentation in *Shank3B^+/−^* and *Cntnap2^−/−^* mice could be related to changes in sniff rate as animals transitioned from being naive to being expert because sniffing modulates neural responses in the olfactory bulb (Verhagen et al., 2007). Sniffing responses were quantified using an airflow sensor (Bolding and Franks, 2018). We analyzed responses to correct no-go responses, to avoid potential influences of licking action (Extended Data Fig. 2-2). Sniff responses to background were also quantified in the 1 s period preceding the onset of the target odor. The average sniff rate (Extended Data Fig. 6-2*A,B*) for the naive *Shank3B^+/−^* and *Cntnap2^−/−^* mice was 1.98 ± 0.05 sniff per second (mean ± SEM, *n* = 504 odor responses, from 4 *Cntnap2^−/−^* mice and 4 *Shank3B^+/−^* mice), and it was not different (*p* = 0.23, double-tailed *t* test, *n* = 8 mice) from the sniff rate in the expert condition (2.02 ± 0.07 sniff per second, mean ± SEM, *n* = 240 trials). The sniff rate did increase in the expert condition, but only after target onset. The average sniff rate in the 1 s after target onset did significantly increase in the expert condition (*p* = 0.038, double-tailed *t* test, *n* = 8 mice) from 2.05 ± 0.05 sniff per second in the naive condition (mean ± SEM, *n* = 504 trials) to 2.46 ± 0.07 sniff per second (mean ± SEM, *n* = 240 trials) in the expert condition.

Baseline sniffing was also not different between the naive and expert conditions. Sniff rate was quantified in the 1 s period preceding the onset of the background odors. The average sniff rate (Extended Data Fig. 6-2) for naive *Shank3B^+/−^* and *Cntnap2^−/−^* mice was 1.96 ± 0.04 sniffs per second (mean ± SEM, *n* = 504 odor responses, 4 *Cntnap2^−/−^* and 4 *Shank3B^+/−^* mice), which was not significantly different (*p* = 0.069, double-tailed *t* test, *n* = 8 mice) from the sniff rate in the expert condition (1.46 ± 0.05 sniffs per second, mean ± SEM, *n* = 240 trials). Changes in sniffing between naive and expert mice occurred only after target onset. Thus, the suppression of background responses in the expert condition compared with the naive condition was not caused by changes in baseline sniffing.

We also tested whether the increased stability of neural responses to targets in expert mice could be explained by reduced trial-to-trial variability in sniffing (Extended Data Fig. 6-2*C*). To test this, we quantified the number of sniffs in the 1 s following target onset on each trial and calculated the coefficient of variation (CV) of the sniff rate, defined as the standard deviation divided by the mean. The CV of sniff rate in naive *Shank3B^+/−^* and *Cntnap2^−/−^* mice was 0.42 ± 0.11 (mean ± SEM, *n* = 8 mice), which was not significantly different (*p* = 0.41, paired *t* test) from that in expert mice (0.46 ± 0.06). Thus, the greater stability of neural responses in expert *Shank3B^+/−^* and *Cntnap2^−/−^* mice was not due to reduced variability in sniffing.

### WT mice responses to background odors were not broadly suppressed with training

In naive WT mice, reduced neural activation during the early background odor period was also associated with improved separability between go and no-go stimuli, although the effect was less pronounced than in the ASD models. Given the limited improvement in odor mixture discriminability associated with early background suppression in WT mice, we asked whether WT mice would exhibit learning-related broad reductions in olfactory bulb responses similar to those observed in *Shank3B^+/−^* and *Cntnap2^−/−^* mice.

WT mice did not show the broad suppression of the background responses with experience as did *Cntnap2^−/−^* and *Shank3B^+/−^* mice (Fig. 6*D*,*E*). The average response to the background in the naive WT mice was 0.90 ± 0.02 *z*-score (mean ± SEM, 2,157 ROI–odor responses), and its average was slightly smaller than the responses in the expert condition (0.98 ± 0.02 *z*-score, mean ± SEM, 1,585 ROI–odor pairs). The mean glomerular activation for individual background odor mixtures in each animal did not show broad suppression. Out of the 16 background odor mixtures measured in 4 *WT* mice in the naive vs expert conditions, only 7 odor mixtures showed suppression in their mean glomerular activation with experience (*p* = 0.40, binomial test), compared with 29 of 31 in our ASD mouse models.

The ratio between average odor responses between expert and naive (Fig. 6*F*) was 0.72 ± 0.18 (mean ± SD, 31 mouse-odor responses) for *Shank3B^+/−^* and *Cntnap2^−/−^* mice, whereas the ratio for WT mice was 1.10 ± 0.43 (mean ± SD, 16 mouse-odor responses). This suppression of background responses in the expert condition was significantly larger for the *Shank3B^+/−^* and *Cntnap2^−/−^* mice compared with the WT mice (*p* = 9.02 × 10^−5^, double-tailed *t* test). The WT responses during the target presentation period were also slightly larger in the expert condition (WT mice expert: 1.88 ± 0.04 *z*-score, mean ± SEM, 1,049 ROI–odor pairs; WT mice naive: 1.44 ± 0.02 *z*-score, mean ± SEM, 1,029 ROI–odor pairs). Reduction of olfactory bulb responses during background presentation after learning was seen only in *Shank3B^+/−^* and *Cntnap2^−/−^* mice, but not in WT mice.

### WT mice sniff rates were not affected by training

We wondered whether the lack of consistent modulation in neural responses in WT mice produced by training would also be accompanied by a lack of modulation in sniffing. The average sniff rate during the background period (Extended Data Fig. 6-3*A,B*) for naive WT mice was 2.83 ± 0.11 sniffs per second (mean ± SEM, *n* = 270 odor responses, 4 WT mice), which was not significantly different (*p* = 0.059, double-tailed paired *t* test, *n* = 4 mice) from that in the expert condition (2.19 ± 0.12 sniffs per second, mean ± SEM, *n* = 149 trials). Sniff rate also did not significantly increase after target onset. In fact, the average rate in the 1 s following target onset decreased slightly, though not significantly (*p* = 0.13, double-tailed *t* test, *n* = 4 mice), from 3.06 ± 0.11 sniffs per second in the naive condition (mean ± SEM, *n* = 270 trials) to 2.45 ± 0.11 sniffs per second in the expert condition (*n* = 149 trials). Baseline sniffing was also unchanged across conditions. The average baseline sniff rate for naive WT mice was 1.60 ± 0.06 sniffs per second (mean ± SEM, *n* = 270 odor responses, 4 WT mice), which was not significantly different (*p* = 0.26, double-tailed *t* test, *n* = 4 mice) from that in the expert condition (1.41 ± 0.07 sniffs per second, mean ± SEM, *n* = 149 trials). There was no consistent modulation in sniffing responses in WT mice with training.

We then asked whether the reliability of sniff responses changed with training in WT mice. Inter-trial variability was quantified using the coefficient of variation (CV) of the sniff rate in the 1 s following target onset, as was done for the ASD mouse models. The CV of sniff rate (Extended Data Fig. 6-3*C*) in the naive condition was 0.43 ± 0.05 (mean ± SEM), which was nearly identical to that in the expert condition (0.43 ± 0.04, *p* = 0.92, paired *t* test). Thus, training also did not alter the reliability of sniff responses in WT mice.

### Naive *Shank3B^+/−^* and *Cntnap2^−/−^* mice did not elevate their sniff rate during odor presentation compared with baseline period

In the naive condition, *Shank3B^+/−^* and *Cntnap2^−/−^* mice showed more variable neural representations of target odors, which became more stable only after training. In contrast, WT mice already exhibited relatively stable neural responses when naive. In other sensory modalities, attention has been shown to reduce variability and stabilize neural activity (Arazi et al., 2019). We therefore asked whether the higher variability observed in *Shank3B^+/−^* and *Cntnap2^−/−^* mice might reflect reduced attention to odors, as mice typically elevate their sniffing to actively explore them (Wesson et al., 2008). Consistent with this idea, prior work has shown that even expert *Cntnap2^−/−^* mice do not elevate their sniff rates in response to odors compared with expert WT mice (Li et al., 2023).

Interestingly, naive *Shank3B^+/−^* and *Cntnap2^−/−^* mice did not elevate their sniffing rates relative to baseline prior to odor onset (Extended Data Fig. 6-2). The baseline sniff rate was 1.96 ± 0.04 sniffs/s (mean ± SEM, *n* = 504 odor responses, 4 *Cntnap2^−/−^* and 4 *Shank3B^+/−^* mice) and remained nearly unchanged during the background period (1.98 ± 0.05 sniffs/s, *p* = 0.46, two-tailed *t* test) as well as during the target period (2.05 ± 0.05 sniffs/s, *p* = 0.86, double-tailed *t* test). Thus, naive ASD model mice showed no elevation in sniff rate in response to odors. With training, however, sniffing became modulated. The baseline sniff rate in expert mice was 1.46 ± 0.05 sniffs/s (*n* = 240 trials) and increased significantly to 2.02 ± 0.07 sniffs/s during the background period (*p* = 9.7 × 10^−4^) and further to 2.46 ± 0.07 sniffs/s during the target period (*p* = 3.05 × 10^−4^).

We wondered whether naive WT mice would also maintain a flat sniff rate or whether they would increase sniffing upon odor delivery. In contrast to the ASD models, naive WT mice did increase their sniff rate relative to baseline (1 s prior to odor onset; Extended Data Fig. 6-3*D,E*). The increase was quantified as the difference between baseline and sniff rate during the background and target periods. Naive WT mice increased their sniff rate by 1.15 ± 0.49 sniffs per second (mean ± SEM, *n* = 4 mice) during the background period and 1.31 ± 0.68 sniffs per second during the target period. These increases were not significantly different from those observed in expert *Shank3B^+/−^* and *Cntnap2^−/−^* mice (0.55 ± 0.10 sniffs per second for background and 0.91 ± 0.14 for target, *n* = 8 mice; *p* = 0.13 and *p* = 0.45, respectively, double-tailed *t* test). We then asked whether WT mice maintained similar increases in sniff rate in the expert condition. WT mice continued to elevate sniffing during both background (0.80 ± 0.49 sniffs per second) and target periods (1.07 ± 0.42 sniffs per second), which were not significantly different from the increases observed in expert *Shank3B^+/−^* and *Cntnap2^−/−^* mice (*p* = 0.51 and *p* = 0.66, respectively, double-tailed *t* test).

Although WT mice did not change their sniff rates in response to odors with training, they already showed elevated sniffing as naive animals, suggesting heightened attention to odors from the outset. In contrast, ASD mouse models increased their sniffing to odors only after training, implying reduced attention to odors in the naive condition. This lack of early attention may contribute to the greater variability observed in their olfactory bulb odor-evoked responses.

## Discussion

With training, both *Shank3B^+/−^* and *Cntnap2^−/−^* mouse models of autism can learn to recognize weak target odors in the presence of strong background odors, transitioning from naive to experts and performing comparably to WT mice. Using wide-field calcium imaging, we found that training improved neural coding in both ASD models and WT mice by enhancing the separation between the neural representations of the go and no-go stimuli. In *Shank3B^+/−^* and *Cntnap2^−/−^* mice, training significantly increased the reliability of neural responses, whereas WT mice, which already exhibited stable representations in the naive state, showed smaller gains. In naive animals, strong activation by background odors degraded neural coding, with a greater impact in the ASD models than in WT mice. Training reduced these large, disruptive background-driven responses in *Shank3B^+/−^* and *Cntnap2^−/−^* mice but had no consistent suppressive effect in WT mice. Thus, our findings reveal a neural mechanism by which performance on a difficult discrimination task can transition from naive to expert states in mouse models of ASD.

We used wide-field calcium imaging to measure neural responses in the olfactory bulb of mouse models of autism using the C57BL/6J-Tg(Thy1-GCaMP6f) GP5.11Dkim/J (Dana et al., 2014). This line expresses GCaMP6f in mitral and tufted cells in the olfactory bulb, as well as in areas that send axons to the olfactory bulb, including the piriform cortex, hippocampus, and entorhinal cortex. Although the raw fluorescence signal includes activity from olfactory bulb neurons as well as bulbar projecting axons from other parts of the brain, feedback inputs representing different odors are diffusely distributed at the spatial scale of individual glomeruli (Boyd et al., 2015) and were substantially reduced by the high-pass spatial filtering used (Extended Data Fig. 1-1). The glomerular signal that we recorded includes a mixture of both mitral and tufted cells which constitute different output channels of the olfactory bulb (Nagayama et al., 2010; Fukunaga et al., 2012; Igarashi et al., 2012), modulated by distinct descending projections into the olfactory bulb (Otazu et al., 2015; Chae et al., 2022).

Behavioral training enhanced both performance and neural discrimination of odor mixtures in *Shank3B^+/−^* and *Cntnap2^−/−^* mice as well as in WT mice. In the ASD models, training increased the reliability of neural responses and reduced activation by background odors, whereas naive WT responses were already as reliable as those of trained mutants and showed no systematic change in background responses. Analysis of sniffing behavior further revealed that naive *Shank3B^+/−^* and *Cntnap2^−/−^* mice did not elevate their sniff rates in response to odors, suggesting reduced attentional engagement. This phenotype parallels findings in children with autism, who show diminished odor-evoked sniff modulation compared with neurotypical children (Rozenkrantz et al., 2015). In contrast, naive WT mice increased their sniffing upon odor presentation, consistent with enhanced attention even before training. Thus, while training improved neural representations in all groups, only the ASD models also acquired enhanced sniffing responses, suggesting that attentional modulation of odor sampling may contribute to the behavioral and neural gains observed in these mice. Although this interpretation highlights the role of attention, it also makes it challenging to directly relate neural discriminability to behavioral performance on individual trials. Because naive and expert conditions differ in attentional state as well as in neural coding, trial-by-trial correlations would be difficult to interpret.

*Shank3* and *Cntnap2* are autism-associated genes with distinct roles in neurons. *Shank3* (Peça et al., 2011) is a scaffold protein that is part of the postsynaptic density whereas *Cntnap2* (Peñagarikano et al., 2011; Selimbeyoglu et al., 2017) is expressed in axons*. Shank3B^+/−^* mice have reduced inhibitory circuitry affecting the striatum (Peça et al., 2011) and the frontal cortex(Guo et al., 2019; Chen et al., 2020), which have been associated with repetitive behaviors as well as social interaction deficits. *Cntnap2^−/−^* mice have also deficits in inhibitory interneurons in neural activation in frontal cortices (Peñagarikano et al., 2011; Selimbeyoglu et al., 2017) as well as broadening of the transmitted action potential resulting in enlarged neurotransmitter release (Scott et al., 2019). Despite differences in gene expression patterns and neural phenotypes between naive *Shank3B^+/−^* and *Cntnap2^−/−^* mice, our findings show that strong neural activation evoked by background odors in the olfactory bulb is associated with less distinct neural representations of go versus no-go targets. This suggests a converging phenotype in the olfactory bulb in mouse models of autism that might extend to other mouse models of ASD. In particular, mouse models of fragile X exhibit olfactory behavioral deficits and may also show unstable odor-evoked responses that could alter neural representations of odors.

It has been hypothesized that large cortical activation produced by reduced cortical inhibition might underlie some of the behavioral symptoms in autism (Rubenstein and Merzenich, 2003 but see also Antoine et al., 2019). Excess cortical activation in primary cortical areas is associated with behavioral errors in auditory (Scott et al., 2022), visual (Ortiz-Cruz et al., 2022), and somatosensory tasks (Guo et al., 2019; Chen et al., 2020). A similar excess activity as has been observed in the cortex in mouse models of ASD might also be present in the olfactory bulb. Our signal includes a mixture of both mitral and tufted cells which constitute different output channels of the olfactory bulb (Nagayama et al., 2010; Fukunaga et al., 2012; Igarashi et al., 2012), modulated by distinct descending projections into the olfactory bulb (Otazu et al., 2015; Chae et al., 2022). The olfactory bulb circuitry might also be sensitive to reduced inhibition because activation of a mitral cell produces large excitatory potentials on itself and other mitral cells, creating long-lasting depolarizations in mitral cell activity through recurrent excitation (Aroniadou-Anderjaska, Ennis, and Shipley, 1999; Carlson, Shipley, and Keller, 2000; Schoppa and Westbrook, 2001; Matsumoto et al., 2009). This recurrent excitation in mitral cells is stabilized by inhibitory interneurons (Gire and Schoppa, 2009) and pharmacological blocking GABA_A_ receptors results in unstable mitral cell activity (Kuruppath et al., 2023). Weaker inhibition in *Shank3B^+/−^* and *Cntnap2^−/−^* mice could potentially result in strong self-sustaining responses that could negatively affect the neural coding by mitral and tufted cells. Large activation produced by novel background odors could act to mask the targets (Rokni et al., 2014). Large activation of mitral and tufted cells could also result in saturation, making it harder for areas downstream of the olfactory bulb to deconvolve the target odors (Adefuin et al., 2022).

Large initial responses produced by background odors were associated with the later large responses of the background and target mixtures in naive *Shank3B^+/−^* and *Cntnap2^−/−^* mice. The initial background responses became smaller as *Shank3B^+/−^* and *Cntnap2^−/−^* mice were trained and performance improved. The reduction in the evoked neural responses in the olfactory bulb could be caused by a reduction in the excitatory drive coming from the olfactory receptors, an increase in the inhibition onto mitral and tufted cells, or a combination of those factors. Presynaptic inhibition on the olfactory receptor neurons axons is controlled through GABA_B_ receptors and can produce large reductions in excitation from olfactory receptors through periglomerular and short axon cells (McGann, 2013). Mitral and tufted cells activity can be modulated by different populations of inhibitory interneurons (Fukunaga et al., 2012; 2014). Descending fibers from anterior olfactory nucleus (Markopoulos et al., 2012; Oettl et al., 2016), piriform cortex (Otazu et al., 2015), as well as neuromodulatory fibers can act on bulbar interneurons, affecting the responses of mitral and tufted cells (Petzold, Hagiwara, and Murthy, 2009; Boyd et al., 2012; Markopoulos et al., 2012; Otazu et al., 2015; Oettl et al., 2016).

Background odors did not only evoke larger activation in naive *Shank3B^−/−^* and *Cntnap2^−/−^* mice, but they produced higher trial-by-trial variability compared with WT mice. Increased variability has been observed in the olfactory bulb of *Shank3B^−/−^* and *Cntnap2^−/−^* knock-out mice (Geramita et al., 2020) and the Fmr1 knock-out (KO) mouse model of fragile X syndrome (Kuruppath et al., 2023). Behavioral deficits in mouse models of ASD have been generally associated with central circuit dysfunction (Selimbeyoglu et al., 2017; Qin et al., 2019; Sacai et al., 2020) but more recent work point toward peripheral nervous system also play a role (Orefice et al., 2019). Our work points toward the olfactory bulb output responses as one of the loci for olfactory deficits in mouse models of ASD.

Our study found that smaller odor responses to background odors in the olfactory bulb output were associated with less overlapping neural representations of the go and the no-go stimuli. Smaller background odor responses in the olfactory bulb of *Shank3B^−/+^* and *Cntnap2^−/−^* mice could be achieved through training/exposure, which in turn improved olfactory behavior. This suggests that one could potentially screen for manipulations that reduce neural activity to help rescue olfactory behavior in mouse models of ASD. Importantly, our approach does not rely on WT mice neural activation as a model for therapeutic development. Instead, it focuses on the patterns of neural activation observed in the autism mouse models themselves, identifying patterns that correlate with improved behavior. In contrast, current strategies for ASD in mouse models typically use WT neural activation as a template, aiming to manipulate neural circuits to make the autism models’ activity resemble that of WT mice activity (Peñagarikano et al., 2015; Walsh et al., 2018; Hörnberg et al., 2020).Our findings may provide a foundation for an alternative approach in therapeutic development that could extend to several mouse models of ASD.

People with ASD have preferences for food odors that they have been exposed to (Luisier et al., 2019). Although the neural mechanisms for enhanced acceptance for exposed odors is not known, our work would point at reduced neural activation on the olfactory bulb as a potential mechanism for reducing the hypersensitivity to smells in ASD (Wicker et al., 2016).

## Synthesis

Reviewing Editor: James Howard, Brandeis University Decisions are customarily a result of the Reviewing Editor and the peer reviewers coming together and discussing their recommendations until a consensus is reached. When revisions are invited, a fact-based synthesis statement explaining their decision and outlining what is needed to prepare a revision will be listed below. The following reviewer(s) agreed to reveal their identity: Praveen Kuruppath, Adam Dewan.

The authors present a well-executed and insightful study examining how odor experience stabilizes glomerular output representations in two mouse models of autism spectrum disorder (ASD). Given the prevalence of abnormal sensory responses in human ASD patients, this topic is of interest and well suited for this journal. To probe the underlying neural mechanisms, the authors recorded olfactory bulb responses in Cntnap2^-/- and Shank3B^-/+ mice during a challenging odor mixture discrimination task involving strong, overlapping background odors-closely resembling real-world sensory complexity. Additionally, they used Thy1-GCaMP6f mice to conduct targeted, high-resolution measurement of mitral and tufted cell activity in the olfactory bulb. Chronic widefield calcium imaging further allowed minimally invasive tracking of these responses during behavior. The authors observed high variable neural responses during the naive state in these ASD models, which mirrors the abnormal sensory responses of individuals with ASD during their initial stimulus interactions. Interestingly, both situations were ameliorated with training/exposure. While the authors tout a foundation for an alternative approach in therapeutic development for ASD, this study is also relevant for olfactory neuroscience and understanding the impact of training on the neural representation of an odor percept. Overall, the reviewers find the study to be a compelling demonstration of new insights into how sensory experience shapes olfactory processing in mouse models of ASD. Below is a detailed list of comments and suggestions aimed at improving the impact and interpretability of the manuscript:

Major Concerns:

1. As noted in the text (lines 94-100), GCaMP6f expression under the Thy1 promoter may include axonal projections from extra-bulbar regions such as piriform cortex, hippocampus, and entorhinal cortex. Although spatial filtering was applied to reduce contamination, residual signals from these projections could confound interpretations attributed specifically to mitral and tufted cell activity. More explicit discussion of how this limitation affects the conclusions would strengthen the manuscript.

2. The fact that analyses were restricted to correct trials raises concerns about the potential impact of trial number on the author's measure of neural variability. While this subsampling of the data will control for performance differences between naive and expert mice, it will also impact the overall variability of the data, as the expert mice will have more correct trials than the naive mice. This is a concern as one of the primary findings is the noticeable reduction of neural variability in the ASD mice with training. The authors should clarify whether they are considering this potential confound, either by matching the number of trials across mice or by some other statistical measure.

3. Mice were trained for approximately five days on the pure odor task prior to background introduction. While this may be sufficient for task acquisition, variability in age (4-10 months) and individual learning rates may affect neural and behavioral outcomes. A more detailed discussion on learning curves and potential age-related effects would provide greater context and robustness.

4. In the discussion, the authors have put forth some interesting hypotheses regarding specific mechanisms that could promote the stabilization of neural activity in the olfactory bulb after training. However, the degree to which the "neural stabilization" hypotheses could be explained by training related changes in sampling behavior (i.e., sniffing) is unclear. This brings about several associated questions:

a. Why is the sniffing rate so low in ASD mice? Given that the authors point to Li et al., 2023 for their training protocol, we assume sniffing was measured in a similar manner. In that paper, sniffing rates during background/target odor presentation are significantly higher. Could the mice be suppressing their sniffing behavior because the odor concentrations for certain odorants are too high?

b. Could the high neural variability of naïve ASD mice be attributed to a lack of attention on the task? Their sniff rates during background/target presentation were similar to baseline (pre odor presentation) in Li et al., 2023. Do ASD mice exhibit an increase sniffing during the background/target odor presentations as compared to baseline?

c. It is expected that mice increase their sniff rate to aid in target detection. This is only observed in the expert ASD mice. Do WT mice follow a similar trend? If WT mice have similar target odor sniffing rates between naïve and expert conditions, it might help explain why their neural activity is more similar between these conditions (as compared to ASD mice).

5. Target odors were delivered at low concentrations (0.025% vapor saturation), activating a limited number of glomeruli (~29.5%), while background odors were presented at higher concentrations (0.1%). This asymmetry may bias the results toward background-dominated responses and could obscure detection of subtle target-evoked signals. The authors should provide additional discussion of, or justification for, this concentration disparity.

6. One of the conclusions from this manuscript relates to the observation that reduced responses to background odors increase the discriminability of neural representations in the olfactory bulb in naïve ASD mice. Since, the authors have collapsed their data across odorants (2 Go odorants, 2 no-go odorants, and 4 background odorants), this leaves open the question of whether this is a universal phenomenon or attributed to a specific odorant of lower volatility that has a reduced masking effect (e.g., ethyl valerate with ethyl propionate). The same question can be extended to target odor identification - do the animals have improved detection of higher volatility odorants (i.e., ethyl propionate)?

Go Target odorants (Assuming I understood the odor delivery correctly):

Isopropyl butyrate = 0.025% SVP, 6mmHg, or ~2 ppm

Propyl butyrate = 0.025% SVP, 5.5mmHg, or ~1.8 ppm

NoGo odorants:

Ethyl propionate = 0.025% SVP, 35.9mmHg, or ~12 ppm

Isobutyl propionate = 0.025% SVP, 6.47mmHg, or ~2 ppm

Background odorants:

Ethyl Butyrate = 0.1% SVP, 12.8mmHg, or ~16.8 ppm

Isoamyl Acetate = 0.1% SVP, 5.6mmHg, or ~7.4 ppm

Ethyl caprcate = 0.1% SVP, 5.7mmHg, or ~7.5 ppm

Ethyl valerate = 0.1% SVP, 4.8mmHg, or ~6.3 ppm

7. The data demonstrate that training improves both behavioral performance and neural discrimination of odor mixtures in Shank3B-/+, Cntnap2-/-, and WT mice. However, it remains unclear whether these improvements stem from enhanced attention, reward-based plasticity, or experience with complex odors. The authors should discuss which of these explanations they consider most likely based on their findings.

8. While group-level effects are evident, trial-by-trial or subject-level correlations between behavioral improvement and neural discriminability are lacking. The authors could discuss whether their data allow such an analysis, or why it may not be feasible in this study.

9. The study reports a reduction in trial-to-trial neural variability with training in ASD models. However, it remains unclear whether similar reductions occur in trained WT animals. The authors should comment on whether they expect this plasticity to be unique to ASD models or a general learning effect.

10. Figure 3 is central to the manuscript's claim regarding reduced neural variability following training. However, it is currently difficult to interpret due to small panel sizes, crowded layout, and low label clarity. Enlarging individual panels, increasing font size, and more clearly delineating comparisons would significantly enhance readability.

11. Figure 4 is overly dense, containing 14 panels (A-M), many of which are complex. While each panel presents relevant information, the visual overload may hinder comprehension. Panels G-J, in particular, would benefit from improved color coding or clearer labeling. Consider splitting this figure into two, grouping related panels (e.g., neural similarity vs. behavioral correlations) for improved clarity.

Minor:

1. Line 158 - % vapor pressure is not correct. The authors should state either % saturated vapor pressure or vapor saturation.

2. Figure 1 - The odor presentations in D could use labels similar to 1B. The panels in C are different sizes.

3. Figure 2 - Known vs variable background odor - should be consistent. Also why is isopropyl blue in color in panel D?

4. While valence can be used to designate the association with reward. This usage in this context is very confusing as odor valence more typically refers to the perceived pleasantness or unpleasantness of an odor, which is not being measured. Please avoid the term "opposite valence" if possible.

## Author Response

15th Aug 2025 Dear Prof. Otazu:

I am pleased to inform you that your manuscript, "Odor experience stabilizes glomerular output representations in two mouse models of autism," has been judged potentially suitable for publication in eNeuro, provided appropriate revisions are made. The decision was a result of the reviewers and me coming together and discussing our recommendations until a consensus was reached. I have put together a fact-based synthesis statement explaining the decision and appended it to this email.

It is important that you revise your manuscript to address the reviewers' concerns and submit a point-by-point reply to each concern. Before making a final decision, one or both reviewers will re-review your revised manuscript.

Your revision must include the manuscript with new text indicated in a bold or highlighted font to aid the re-review of the manuscript. Separately, please provide a clean copy of the manuscript that includes the title page, which will be published if accepted. Please closely review your manuscript at this time for any final corrections in style or substance. Please consult our Revised Submission Checklist for full details on preparing your revision: https://www.eneuro.org/content/revising-finalizing-manuscript#revisions In an effort to improve rigor, transparency, and reproducibility, eNeuro strongly encourages the use of estimation statistics. If the use of estimation statistics applies to your research, you may consider including it in your revision. More details and resources can be found in the Information for Authors: https://www.eneuro.org/content/preparing-manuscript#statistics Your submission must include publication-quality figures, each in a separate EPS or TIFF (300 dpi) file. Please make sure your figures adhere to style requirements to avoid delays in manuscript processing. Detailed guidelines for figures are available in our Information for Authors: https://www.eneuro.org/content/preparing-manuscript#figures When uploading the revised manuscript, the corresponding author will need to complete the electronic License to Publish form; if not completed during submission, a link to the form will also be available on the author's home page at https://eneuro.msubmit.net.

All authors are invited to submit visually appealing, high-quality images to be used to promote their article, including on the journal homepage. Please submit your images, as well as an image caption and credit information, directly to the peer review system with your revision. Additional details on sizing and image specifications can be found in the Information for Authors: https://www.eneuro.org/content/revising-finalizing-manuscript#image Please return your revision within 3 months of this decision. When you are ready to submit your revision, you can log in using the link below and click on the manuscript number to create your revision. If you have any questions or concerns, or need more time to prepare your revision, please contact eneuro@sfn.org. https://eneuro.msubmit.net/cgi-bin/main.plex Yours sincerely, James Howard Reviewing Editor eNeuro -------------------------------------------- Manuscript Instructions --------------------------------------------- Synthesis of Reviews:

Computational Neuroscience Model Code Accessibility Comments for Author (Required):

N/A Synthesis Statement for Author (Required):

The authors present a well-executed and insightful study examining how odor experience stabilizes glomerular output representations in two mouse models of autism spectrum disorder (ASD). Given the prevalence of abnormal sensory responses in human ASD patients, this topic is of interest and well suited for this journal. To probe the underlying neural mechanisms, the authors recorded olfactory bulb responses in Cntnap2^-/- and Shank3B^-/+ mice during a challenging odor mixture discrimination task involving strong, overlapping background odors-closely resembling real-world sensory complexity. Additionally, they used Thy1-GCaMP6f mice to conduct targeted, high-resolution measurement of mitral and tufted cell activity in the olfactory bulb. Chronic widefield calcium imaging further allowed minimally invasive tracking of these responses during behavior. The authors observed high variable neural responses during the naive state in these ASD models, which mirrors the abnormal sensory responses of individuals with ASD during their initial stimulus interactions. Interestingly, both situations were ameliorated with training/exposure. While the authors tout a foundation for an alternative approach in therapeutic development for ASD, this study is also relevant for olfactory neuroscience and understanding the impact of training on the neural representation of an odor percept. Overall, the reviewers find the study to be a compelling demonstration of new insights into how sensory experience shapes olfactory processing in mouse models of ASD.

We thank the reviewers for their appreciation of our work, We have addressed their concerns with more analysis of the imaging and the sniffing data. We are thankful for their suggestions regarding the lack of attentional modulation in mouse models of autism, which we have confirmed by analyzing the sniffing data. We are also thankful for their multiple suggested controls that have improved the robustness of our results as well as the ideas to improve the clarity of our manuscript.

Below is a detailed list of comments and suggestions aimed at improving the impact and interpretability of the manuscript:

Major Concerns:

1. As noted in the text (lines 94-100), GCaMP6f expression under the Thy1 promoter may include axonal projections from extra-bulbar regions such as piriform cortex, hippocampus, and entorhinal cortex. Although spatial filtering was applied to reduce contamination, residual signals from these projections could confound interpretations attributed specifically to mitral and tufted cell activity. More explicit discussion of how this limitation affects the conclusions would strengthen the manuscript.

We thank the reviewer for this comment. We have added the following paragraph to the discussion discussing this limitation of the widefield imaging (page 15):

We used widefield calcium imaging to measure neural responses in the olfactory bulb of mouse models of autism using the C57BL/6J-Tg(Thy1-GCaMP6f) GP5.11Dkim/J (Dana et al. 2014). This line expresses GCaMP6f in mitral and tufted cells in the olfactory bulb, as well as in areas that send axons to the olfactory bulb, including the piriform cortex, hippocampus, and entorhinal cortex. Although the raw fluorescence signal includes activity from olfactory bulb neurons as well as bulbar projecting axons from other parts of the brain, feedback inputs representing different odors are diffusely distributed at the spatial scale of individual glomeruli(Boyd et al. 2015) and were substantially reduced by the high-pass spatial filtering used (see Figure 1-1). The glomerular signal that we recorded includes a mixture of both mitral and tufted cells which constitute different output channels of the olfactory bulb(Fukunaga et al. 2012; Nagayama et al. 2010; Igarashi et al. 2012), modulated by distinct descending projections into the olfactory bulb(Otazu et al. 2015; Chae et al. 2022).

2. The fact that analyses were restricted to correct trials raises concerns about the potential impact of trial number on the author's measure of neural variability. While this subsampling of the data will control for performance differences between naive and expert mice, it will also impact the overall variability of the data, as the expert mice will have more correct trials than the naive mice. This is a concern as one of the primary findings is the noticeable reduction of neural variability in the ASD mice with training. The authors should clarify whether they are considering this potential confound, either by matching the number of trials across mice or by some other statistical measure.

We thank the reviewer for this important control. We have added the following section to include an analysis that matched the number of trials between the naïve and the expert conditions as follows (Page 8):

Differences in the number of repeats between the naïve and expert conditions could potentially bias the estimation of variability. To test whether our conclusions were robust to this factor, we determined, for each animal and each odor mixture, the number of trials available in the naïve and expert conditions. We then recalculated the coefficient of variation using the same number of trials in both conditions by randomly subsampling trials from the condition with more repeats to match the other. Even after this control, variability remained higher in the naïve condition (coefficient of variation = 1.52 {plus minus} 0.04, mean {plus minus} s.e.m., n = 1099 glomerulus-odor pairs) compared to the expert condition (1.04 {plus minus} 0.03, mean {plus minus} s.e.m., n = 1108 glomerulus-odor pairs, p = 0.0019, double-tailed t-test,,n=4 Cntnap2-/- mice and 3 Shank3B+/- mice; see Figure 3E).

3. Mice were trained for approximately five days on the pure odor task prior to background introduction. While this may be sufficient for task acquisition, variability in age (4-10 months) and individual learning rates may affect neural and behavioral outcomes. A more detailed discussion on learning curves and potential age-related effects would provide greater context and robustness.

We thank the reviewer for this comment. The most variable result was the behavioral responses in the naïve conditions. Some animals did better than others. The reviewers are right that part of this variability was due to the difference in ages in the mice. However, the difference disappeared in the expert condition (Page 6): "There was higher variability in behavioral performance in the naïve condition across mice (see Figure 2-1). This variability was partially explained by age, with younger mice performing better. Specifically, performance in the naïve condition was negatively correlated with age (r = -0.72, p = 0.009, n = 12 mice). However, this effect of age was no longer present in expert condition, where the correlation between age and performance was not significant (p = 0.12).

4. In the discussion, the authors have put forth some interesting hypotheses regarding specific mechanisms that could promote the stabilization of neural activity in the olfactory bulb after training. However, the degree to which the "neural stabilization" hypotheses could be explained by training related changes in sampling behavior (i.e., sniffing) is unclear.

The reviewers raise an important point. In the revised version of the manuscript we have tested whether the variability in the sniffing response might be potentially causing the changes in neural variability between the naïve and the expert condition (page 12):

We also tested whether the increased stability of neural responses to targets in expert mice could be explained by reduced trial-to-trial variability in sniffing (see Figure 6-2C). To test this, we quantified the number of sniffs in the 1 s following target onset on each trial and calculated the coefficient of variation (CV) of the sniff rate, defined as the standard deviation divided by the mean. The CV of sniff rate in naïve Shank3B+/- and Cntnap2-/- mice was 0.42 {plus minus} 0.11 (mean {plus minus} s.e.m., n = 8 mice), which was not significantly different (p = 0.41, paired t-test) from that in expert mice (0.46 {plus minus} 0.06). Thus, the greater stability of neural responses in expert Shank3B+/- and Cntnap2-/- mice was not due to reduced variability in sniffing.

This brings about several associated questions: a. Why is the sniffing rate so low in ASD mice? Given that the authors point to Li et al., 2023 for their training protocol, we assume sniffing was measured in a similar manner. In that paper, sniffing rates during background/target odor presentation are significantly higher. Could the mice be suppressing their sniffing behavior because the odor concentrations for certain odorants are too high? b. Could the high neural variability of naïve ASD mice be attributed to a lack of attention on the task? Their sniff rates during background/target presentation were similar to baseline (pre odor presentation) in Li et al., 2023. Do ASD mice exhibit an increase sniffing during the background/target odor presentations as compared to baseline? We thank the reviewer for these points. Li et al., 2023 reported the sniffing responses in well trained Cntnap2 mice and they were found to have smaller elevations of their sniff rate compared to WT mice. In the naïve mouse models of ASD, there was essentially no elevation of the sniff rate in response to the odors. We do think that the lack of sniff increase in response to odors is consistent with lack of attention in the naïve mouse models of ASD. Attention can stabilize neural responses in other modalities.

We have added the following section (page 14):

In the naïve condition, Shank3B+/- and Cntnap2-/- mice showed more variable neural representations of target odors, which became more stable only after training. By contrast, WT mice already exhibited relatively stable neural responses when naïve. In other sensory modalities, attention has been shown to reduce variability and stabilize neural activity (Arazi et al., 2019). We therefore asked whether the higher variability observed in Shank3B+/- and Cntnap2-/- mice might reflect reduced attention to odors, as mice typically elevate their sniffing to actively explore them (Wesson et al., 2008). Consistent with this idea, prior work has shown that even expert Cntnap2-/- mice do not elevate their sniff rates in response to odors compared to expert WT mice (Li et al. 2023) . "Interestingly, naïve Shank3B+/- and Cntnap2-/- mice did not elevate their sniffing rates relative to baseline prior to odor onset (see Figure 6-2). The baseline sniff rate was 1.96 {plus minus} 0.04 sniffs/s (mean {plus minus} s.e.m., n = 504 odor responses, 4 Cntnap2-/- and 4 Shank3B+/- mice) and remained nearly unchanged during the background period (1.98 {plus minus} 0.05 sniffs/s, p = 0.46, two-tailed t-test) as well as during the target period (2.05 {plus minus} 0.05 sniffs/s, p = 0.86, double tailed t-test). Thus, naïve ASD model mice showed no elevation in sniff rate in response to odors. With training, however, sniffing became modulated. The baseline sniff rate in expert mice was 1.46 {plus minus} 0.05 sniffs/s (n = 240 trials) and increased significantly to 2.02 {plus minus} 0.07 sniffs/s during the background period (p = 9.7 × 10⁻⁴) and further to 2.46 {plus minus} 0.07 sniffs/s during the target period (p = 3.05 × 10⁻⁴). c. It is expected that mice increase their sniff rate to aid in target detection. This is only observed in the expert ASD mice. Do WT mice follow a similar trend? If WT mice have similar target odor sniffing rates between naïve and expert conditions, it might help explain why their neural activity is more similar between these conditions (as compared to ASD mice).

There was no change in the sniff rates between the naïve and the expert WT mice (page 13):

WT mice sniff rates were not affected by training We wondered whether the lack of consistent modulation in neural responses in WT mice produced by training would also be accompanied by a lack of modulation in sniffing. The average sniff rate during the background period (see Figure 6-3AB) for naïve WT mice was 2.83 {plus minus} 0.11 sniffs per second (mean {plus minus} s.e.m., n = 270 odor responses, 4 WT mice), which was not significantly different (p = 0.059, double-tailed paired t-test, n = 4 mice) from that in the expert condition (2.19 {plus minus} 0.12 sniffs per second, mean {plus minus} s.e.m., n = 149 trials). Sniff rate also did not significantly increase after target onset. In fact, the average rate in the 1 s following target onset decreased slightly, though not significantly (p = 0.13, double-tailed t-test, n = 4 mice), from 3.06 {plus minus} 0.11 sniffs per second in the naïve condition (mean {plus minus} s.e.m., n = 270 trials) to 2.45 {plus minus} 0.11 sniffs per second in the expert condition (n = 149 trials). Baseline sniffing was also unchanged across conditions. The average baseline sniff rate for naïve WT mice was 1.60 {plus minus} 0.06 sniffs per second (mean {plus minus} s.e.m., n = 270 odor responses, 4 WT mice), which was not significantly different (p = 0.26, double-tailed t-test, n = 4 mice) from that in the expert condition (1.41 {plus minus} 0.07 sniffs per second, mean {plus minus} s.e.m., n = 149 trials). There was no consistent modulation in sniffing responses in WT mice with training.

We then asked whether the reliability of sniff responses changed with training in WT mice. Inter-trial variability was quantified using the coefficient of variation (CV) of the sniff rate in the 1 s following target onset, as was done for the ASD mouse models. The CV of sniff rate (see Figure 6-3C) in the naïve condition was 0.43 {plus minus} 0.05 (mean {plus minus} s.e.m.), which was nearly identical to that in the expert condition (0.43 {plus minus} 0.04, p = 0.92, paired t-test). Thus, training also did not alter the reliability of sniff responses in WT mice.

Interestingly, the WT mice did elevate their sniff rates in response to odors starting from the naïve condition (page 14):

We wondered whether naïve WT mice would also maintain a flat sniff rate or whether they would increase sniffing upon odor delivery. In contrast to the ASD models, naïve WT mice did increase their sniff rate relative to baseline (1 s prior to odor onset, see Figure 6-3DE). The increase was quantified as the difference between baseline and sniff rate during the background and target periods. Naïve WT mice increased their sniff rate by 1.15 {plus minus} 0.49 sniffs per second (mean {plus minus} s.e.m., n = 4 mice) during the background period and 1.31 {plus minus} 0.68 sniffs per second during the target period. These increases were not significantly different from those observed in expert Shank3B+/- and Cntnap2-/- mice (0.55 {plus minus} 0.10 sniffs per second for background and 0.91 {plus minus} 0.14 for target, n = 8 mice; p = 0.13 and p = 0.45, respectively, double-tailed t-test). We then asked whether WT mice maintained similar increases in sniff rate in the expert condition. WT mice continued to elevate sniffing during both background (0.80 {plus minus} 0.49 sniffs per second) and target periods (1.07 {plus minus} 0.42 sniffs per second), which were not significantly different from the increases observed in expert Shank3B+/- and Cntnap2-/- mice (p = 0.51 and p = 0.66, respectively, double-tailed t-test).

5. Target odors were delivered at low concentrations (0.025% vapor saturation), activating a limited number of glomeruli (~29.5%), while background odors were presented at higher concentrations (0.1%). This asymmetry may bias the results toward background-dominated responses and could obscure detection of subtle target-evoked signals. The authors should provide additional discussion of, or justification for, this concentration disparity.

We used strong background odors with weak target odors to increase the difficulty of the task. We have added the following section (page 5):

The weak target odors (0.025% vapor saturation) activated a smaller fraction of glomeruli (29.5% {plus minus} 2.0%, mean {plus minus} s.d) compared to the stronger (0.1% vapor saturation) background odors (43.5% {plus minus} 6.2%, mean {plus minus} s.d) as previously determined using intrinsic imaging. The glomeruli activated by the background odors overlapped with the target odors, resulting in effective masking and increasing the difficulty of the task (Li et al. 2023).

6. One of the conclusions from this manuscript relates to the observation that reduced responses to background odors increase the discriminability of neural representations in the olfactory bulb in naïve ASD mice. Since, the authors have collapsed their data across odorants (2 Go odorants, 2 no-go odorants, and 4 background odorants), this leaves open the question of whether this is a universal phenomenon or attributed to a specific odorant of lower volatility that has a reduced masking effect (e.g., ethyl valerate with ethyl propionate). The same question can be extended to target odor identification - do the animals have improved detection of higher volatility odorants (i.e., ethyl propionate)? Go Target odorants (Assuming I understood the odor delivery correctly):

Isopropyl butyrate = 0.025% SVP, 6mmHg, or ~2 ppm Propyl butyrate = 0.025% SVP, 5.5mmHg, or ~1.8 ppm NoGo odorants:

Ethyl propionate = 0.025% SVP, 35.9mmHg, or ~12 ppm Isobutyl propionate = 0.025% SVP, 6.47mmHg, or ~2 ppm Background odorants:

Ethyl Butyrate = 0.1% SVP, 12.8mmHg, or ~16.8 ppm Isoamyl Acetate = 0.1% SVP, 5.6mmHg, or ~7.4 ppm Ethyl caprcate = 0.1% SVP, 5.7mmHg, or ~7.5 ppm Ethyl valerate = 0.1% SVP, 4.8mmHg, or ~6.3 ppm The reviewer asks an important question whether the reduction of background responses with training would be limited to the odors with low vapor pressure. We have tested this hypothesis, and we did not find systematic effects of the background nor the target vapor pressures (page 10): "In naïve Shank3B-/+ and Cntnap2-/- mice, spontaneous fluctuations in background odor responses influenced the fidelity of target representation. The four background odors used in this study had vapor pressures ranging from 4.8 mmHg (ethyl valerate) to 12.8 mmHg (ethyl butyrate). We asked whether the improved neural discriminability observed in trials with lower background activation was specific to low vapor pressure odors. To quantify this, we measured coding improvement as the change in similarity to the nearest opposite-value mixture between low and high background response trials. Across animals and odors, coding improvement was not correlated with the vapor pressure of the background odors (R = -0.04, p = 0.77, see Figure 5-1) or of the target odors (R = -0.01, p = 0.95)." 7. The data demonstrate that training improves both behavioral performance and neural discrimination of odor mixtures in Shank3B-/+, Cntnap2-/-, and WT mice. However, it remains unclear whether these improvements stem from enhanced attention, reward-based plasticity, or experience with complex odors. The authors should discuss which of these explanations they consider most likely based on their findings.

We thank the reviewers for their suggestions to analyze more thoroughly the sniffing data and to test whether the mouse models of ASD might not be attending to the odors in the naïve condition. Given this new and interesting result, we have added the following paragraph to the discussion (page 15): "Behavioral training enhanced both performance and neural discrimination of odor mixtures in Shank3B+/- and Cntnap2-/- mice as well as in WT mice. In the ASD models, training increased the reliability of neural responses and reduced activation by background odors, whereas naïve WT responses were already as reliable as those of trained mutants and showed no systematic change in background responses. Analysis of sniffing behavior further revealed that naïve Shank3B+/- and Cntnap2-/- mice did not elevate their sniff rates in response to odors, suggesting reduced attentional engagement. This phenotype parallels findings in children with autism, who show diminished odor-evoked sniff modulation compared to neurotypical children (Rozenkrantz et al., 2015). By contrast, naïve WT mice increased their sniffing upon odor presentation, consistent with enhanced attention even before training. Thus, while training improved neural representations in all groups, only the ASD models also acquired enhanced sniffing responses, suggesting that attentional modulation of odor sampling may contribute to the behavioral and neural gains observed in these mice." 8. While group-level effects are evident, trial-by-trial or subject-level correlations between behavioral improvement and neural discriminability are lacking. The authors could discuss whether their data allow such an analysis, or why it may not be feasible in this study.

In this study, we compared neural responses between the naïve and expert conditions. However, these conditions also differ in attentional state, making it difficult to disentangle the contributions of attention from changes in neural representation. As a result, trial-by-trial correlations between neural discriminability and behavioral performance would be confounded by the attentional effect and are not ideally suited to address this question with our data. We have added the following paragraph to the discussion (page 16): " Although this interpretation highlights the role of attention, it also makes it challenging to directly relate neural discriminability to behavioral performance on individual trials. Because naïve and expert conditions differ in attentional state as well as in neural coding, trial-by-trial correlations would be difficult to interpret.

9. The study reports a reduction in trial-to-trial neural variability with training in ASD models. However, it remains unclear whether similar reductions occur in trained WT animals. The authors should comment on whether they expect this plasticity to be unique to ASD models or a general learning effect.

In the revised version of the manuscript, we have tested whether there was a reduction in neural variability with training in WT mice. We did not find a difference, with the naïve WT mice having as reliable neural response as the trained mouse models of ASD (page 8): "The target odor responses in naïve WT mice (n=4) were as reliable as the Shank3B-/+ and Cntnap2-/- mice following training (see Figure 3C-D). Naive WT mice CV was 1.14{plus minus}0.03 (4 WT mice, mean{plus minus}s.e.m, n=1082 glomerulus-odor pairs) similar to the CV of the expert Shank3B+/- and Cntnap2-/- mice (1.06{plus minus}0.03 ,mean{plus minus}s.e.m, n= 1323 glomerulus-odor pairs). The differences of the average CV per animal was not significantly different (p= 0.95, double tailed t-test) between naïve WT mice (1.12 {plus minus}0.22, mean{plus minus}st.d, n=4 WT mice) and the expert mouse models of ASD (1.13{plus minus} 0.19, mean{plus minus}st.d, n=7 mice , 4 Cntnap2-/- and 3 Shank3B-/+ mice). In the WT mice, there was no significant increase (p=0.69, double tailed paired t-test) in average reliability with training with a CV coefficient of variation of 1.01{plus minus} 0.03 after training (mean{plus minus}s.e.m, n= 1133 glomerulus-odor pairs). " 10. Figure 3 is central to the manuscript's claim regarding reduced neural variability following training. However, it is currently difficult to interpret due to small panel sizes, crowded layout, and low label clarity. Enlarging individual panels, increasing font size, and more clearly delineating comparisons would significantly enhance readability.

We have edited Figure 3 and we have made it more clear the comparisons between naïve and expert conditions for variability in the mouse models of ASD as well as comparisons of variability between expert mouse models of ASD and naïve WT mice.

Figure 3: Behavioral training reduced the variability of neural representations in the olfactory bulb in Shank3B-/+ and Cntnap2-/- mice. A. Example of the response of a glomerulus of a Cntnap2-/- mouse to a no-go mixture in the naïve condition, showing variable responses to different presentations of the mixture. B. Response of a glomerulus in the expert condition of the same Cntnap2-/- mouse, showing less trial-by-trial variability. C. Example of a stable response of a glomerulus in a naïve WT mice. D. Neural responses per glomeruli per trial were quantified as the average response in the 1 s following the target onset for correct rejections. The coefficient of variation was calculated for each roi-odor per mice and is shown in the bar graphs. The average coefficient of variation per mice is shown in individual symbols was reduced in Shank3B-/+ and Cntnap2-/- mice in the expert condition. Coefficient of variation in naïve WT mice was similar to the already trained mouse models of ASD. E. Coefficient of variation calculated using the same number of trials between naïve and expert mouse models of ASD show similar increase in reliability in the expert condition.

11. Figure 4 is overly dense, containing 14 panels (A-M), many of which are complex. While each panel presents relevant information, the visual overload may hinder comprehension. Panels G-J, in particular, would benefit from improved color coding or clearer labeling. Consider splitting this figure into two, grouping related panels (e.g., neural similarity vs. behavioral correlations) for improved clarity.

We thank the reviewers for this suggestion that helps clarify the paper. We have separated the figure 4 into 2. One figure is relating to the effect of elevated evoked background activity on the subsequent activity produced by the target and the odor. The second figures relates to the effects of elevated background activity on the neural discriminability of the target. We have also added more clear labeling:

Figure 4: Fluctuations in background odors responses affected responses during the target odor presentation in naive Shank3B-/+ and Cntnap2-/- mice r. A. Responses to the same odor mixtures were analyzed on individual presentations. B. Example of a single glomerulus responses in a Cntnap2-/- mouse response showing a trial where the background odor produced a large response and there was also a large response during the target and background period. C. Trial where the same background (as in B) produced a small response and there was also a small response during the target and background period. D. Example showing responses of a single glomerulus to multiple repeats of the same backgrounds and target combination. E. Example of the strong linear correlation between the response in the 1 s period preceding the onset of the target and the 1 s after the target onset for a single ROI in a Cntnap2-/- mouse. Individual circles represent individual trials. F. There was strong linear correlation between the 1 s response to the target and background mixture and the background responses preceding the target for Shank3B-/+ and Cntnap2-/- mice. Background responses were evaluated in a 1 s sliding window. The blue line is the average correlation across all animals.

- Figure 5: Fluctuations in background odors in naive Shank3B-/+ and Cntnap2-/- mice reduced the discriminability of neural representations.. A-B. We separated trials in the naïve condition based on the background odor response as high background or low background. Example of population neural responses in a Shank3B-/+ mouse to the same mixture for a high background and a low background response, where each odor matched with closest match of the opposite value. Population neural responses were larger and more similar to its closest match of the opposite value in the high background condition compared to the low background condition. C-D. For each target and background mixture, we calculated the normalized dot product with the target and odor backgrounds of the opposite value and determined the closest match of the opposite value for both the high background condition and the low background condition. E. Closest match of the opposite value in the high background versus the low background condition for 64 target and background odor mixture from 4 Cntnap2-/- and 4 Shan3B+/- mice, and 8 odor mixtures. For most of the mixtures there was a decrease in similarity to the closest match of the opposite value in the low background condition compared to the high background condition. F. Closest match of the opposite value in the high background versus the low background condition for 32 target and background odor mixture from 4 WT mice. There was also a decrease in similarity to the closest match of the opposite value in the low background condition compared to the high background condition. G. Coding improvement was defined as the reduction in similarity to the nearest neighbor of the opposite value seen with low background activation. This coding improvement was larger for mouse models of ASD. Symbols represent individual mice coding improvement averaged over all odor mixtures.

Minor:

1. Line 158 - % vapor pressure is not correct. The authors should state either % saturated vapor pressure or vapor saturation.

We have corrected this as well as all other instances to clarify that we refer to % of saturated vapor pressure.

2. Figure 1 - The odor presentations in D could use labels similar to 1B. The panels in C are different sizes.

We have added the labels to figure 1D. We have also made the panels in C of the same size.

3. Figure 2 - Known vs variable background odor - should be consistent. We have made the labelling consistent.

Also why is isopropyl blue in color in panel D? We have fixed the error in the color. We apologize for this.

4. While valence can be used to designate the association with reward. This usage in this context is very confusing as odor valence more typically refers to the perceived pleasantness or unpleasantness of an odor, which is not being measured. Please avoid the term "opposite valence" if possible.

We thank the reviewers for this suggestion. We have changed the wording in the figure and the text from odor valence to odor value, to avoid confusion.
