# Supplementary figures and images for: Odor Experience Stabilizes Glomerular Output Representations in Two Mouse Models of Autism
Source: eNeuro. 2025 Oct 28;12(10):ENEURO.0271-25.2025. doi: 10.1523/ENEURO.0271-25.2025 (PMC12572760; doi:10.1523/ENEURO.0271-25.2025)

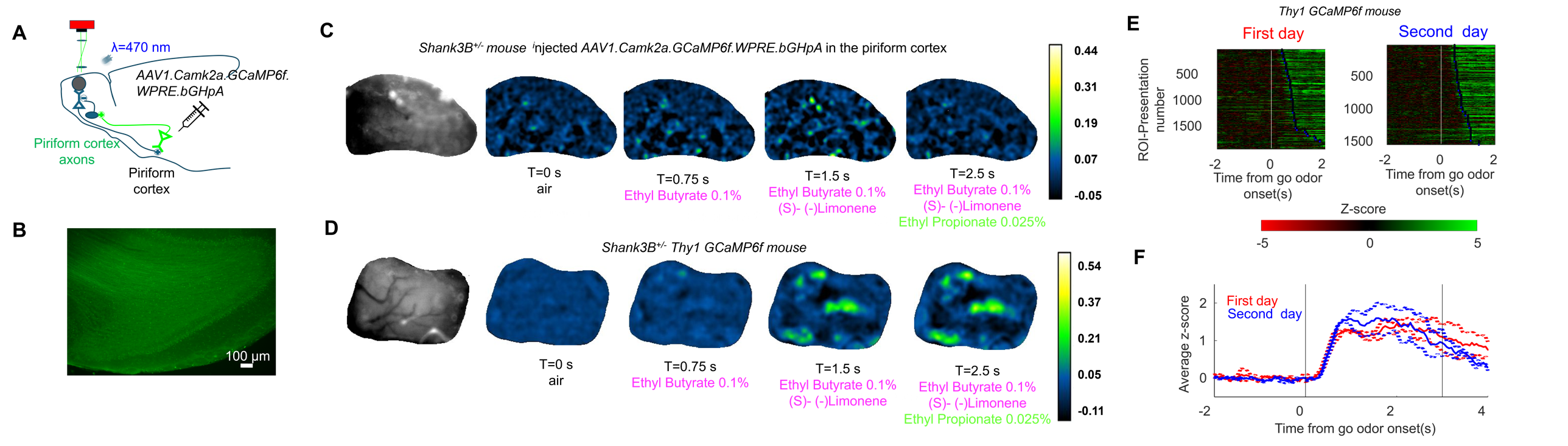

Supplement: Figure 1-1 — Widefield fluorescence reflects activity from neurons in the olfactory bulb. A. We injected AAV virus to express GCaMP6f in the piriform cortex of a Shank3B+/- mouse. After two weeks, we imaged the axons using thinned bone technique as we used with the Thy1-GCaMP6f mice. B. Histology section showing strong expression of GCaMP6f mice in axons from piriform cortex in the olfactory bulb. C-D. Examples of widefield images in response to odors did not reveal glomerular structures in the virus injected mouse, but they were evident in the Thy1-GCaMP6f mouse. E-F. GCaMP6f is expressed in the soma and the dendrites of mitral and tufted cells (see Figure 1C), so widefield calcium signal could potentially reflect activity from these two cell populations. Two-photon microscopy has revealed that mitral cells somatic calcium responses in WT mice in response to pure go-target odors (S+ stimulus) are suppressed by reward association, whereas calcium responses in the more superficial tufted cells do not. In addition, calcium signals from mitral cell apical dendrites are not suppressed by reward association (Lindeman et al. 2024). To determine whether the widefield calcium signal reflected the mitral cells somatic responses, we analyzed the widefield responses as 2 Thy1-GCaMP6f WT mice that learned to associate target odors in our go/no-go paradigm as this condition matches the go/no-go behavior used in Lindeman et al. The WT mice were already trained to discriminate a first odor pair, that is to lick in response to isopropyl butyrate and to refrain from licking for isobutyl propionate both presented at a low concentration of 0.025% of saturated vapor pressure. On the first day, the animals were presented with a novel pair of target odors with propyl butyrate (S+) and ethyl propionate (S-) at 0.025% of saturated vapor pressure. The behavioral performance on the novel odor pair on the first day was close to chance levels (52.3%, 306 trials). The novel odor pair was presented again on [file eneuro-12-ENEURO.0271-25.2025-s007.tif]

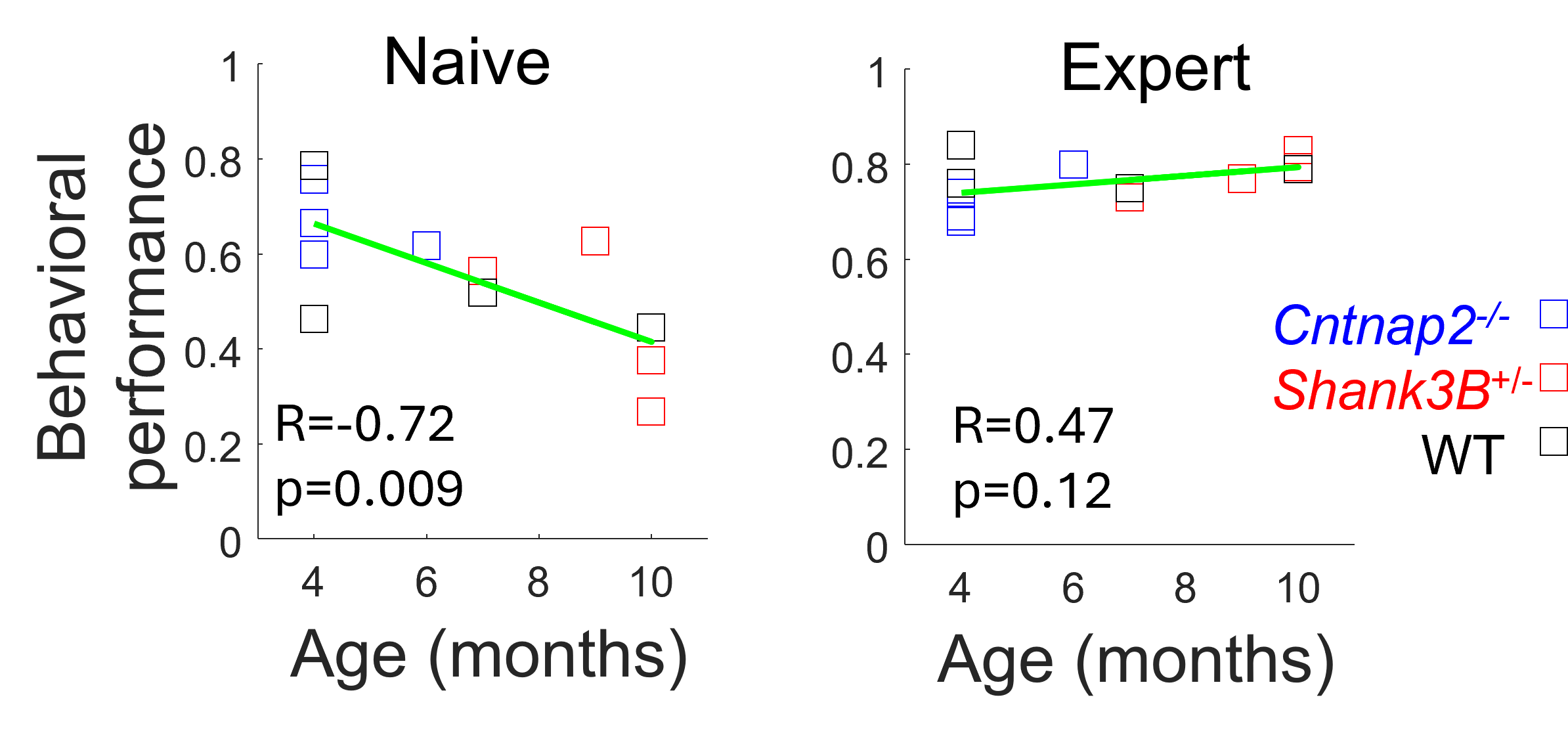

Supplement: Figure 2-1 — Behavioral performance and age. Younger mice performed better in the naïve condition compared to older mice. The difference disappeared in the expert condition. Each symbol is an individual mouse. Download Figure 2-1, TIF file. [file eneuro-12-ENEURO.0271-25.2025-s006.tif]

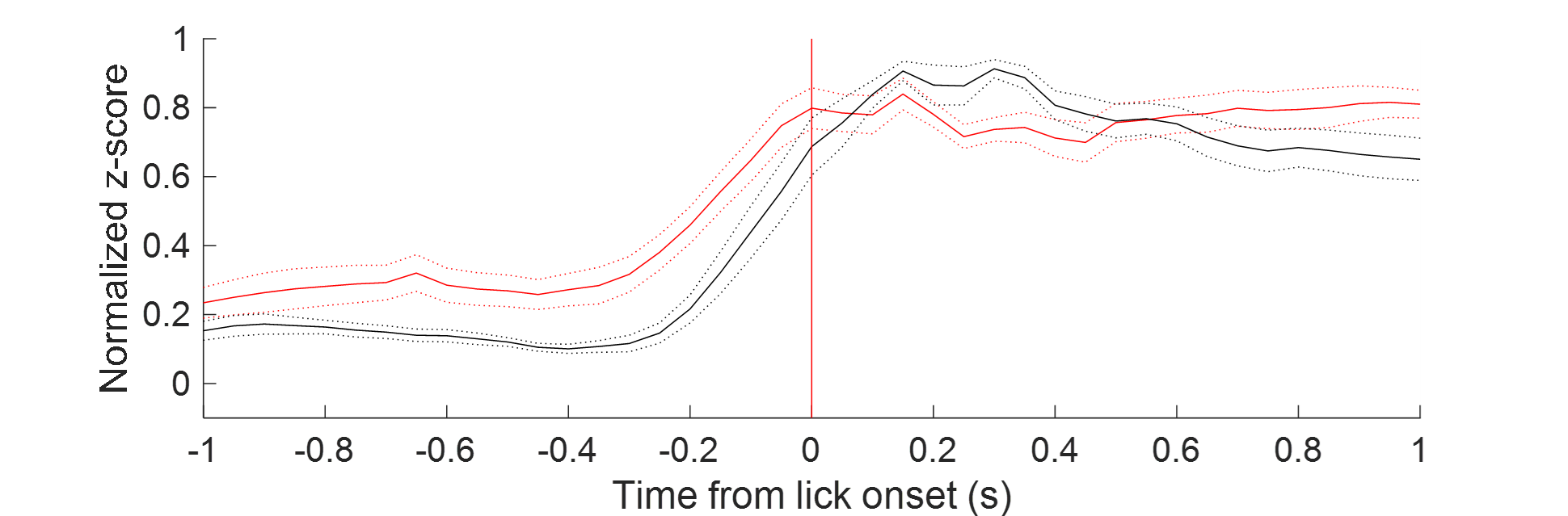

Supplement: Figure 2-2 — Glomerular responses precede the licking action. Average glomerular responses for Shank3B+/- and Cntnap2-/- mice for hits with background odors aligned to the first lick, as measured as the electrical contact with the licking tube. An average licking response was calculated for each session (12 sessions for the Shank3B+/- mice and nine sessions for the Cntnap2-/- mice). Lines are mean ± s.e.m. There was an elevation in the neural response >200 ms that preceded the licking response. Download Figure 2-2, TIF file. [file eneuro-12-ENEURO.0271-25.2025-s005.tif]

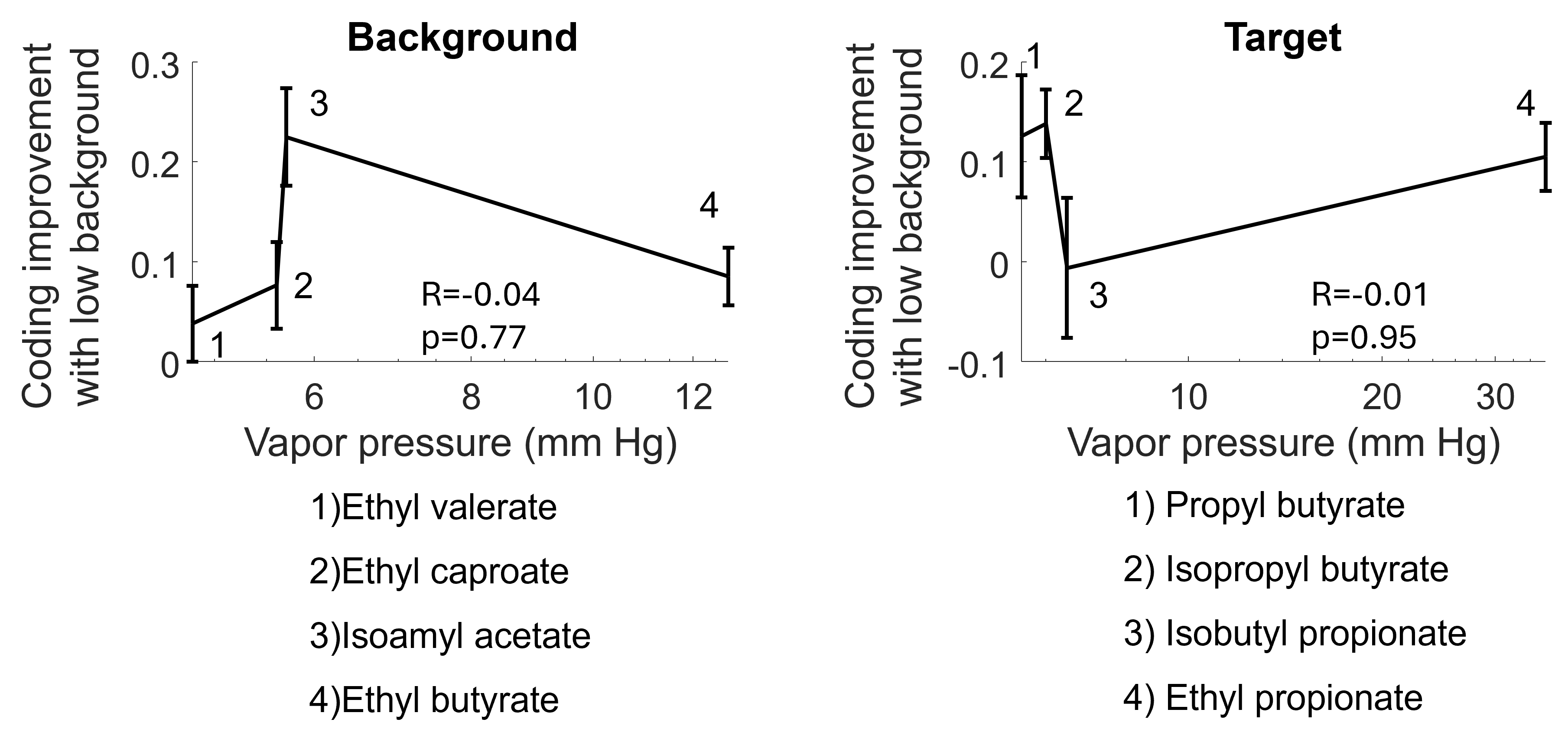

Supplement: Figure 5-1 — Coding improvement produced by lower background activation did not depend on the background nor target odor vapor pressure in Cntnap2-/- and Shank3B+/- mice. Coding improvement was defined as the reduction in similarity to the nearest neighbor of the opposite value seen with low background activation. Each error bar is the mean ± s.em of average coding improvement for the background odors and target odors for all 4 Cntnap2-/- and 4 Shank3B+/- mice. There was no monotonic relationship between the vapor pressure and the coding improvement produced in trials with reduced background activation. Download Figure 5-1, TIF file. [file eneuro-12-ENEURO.0271-25.2025-s004.tif]

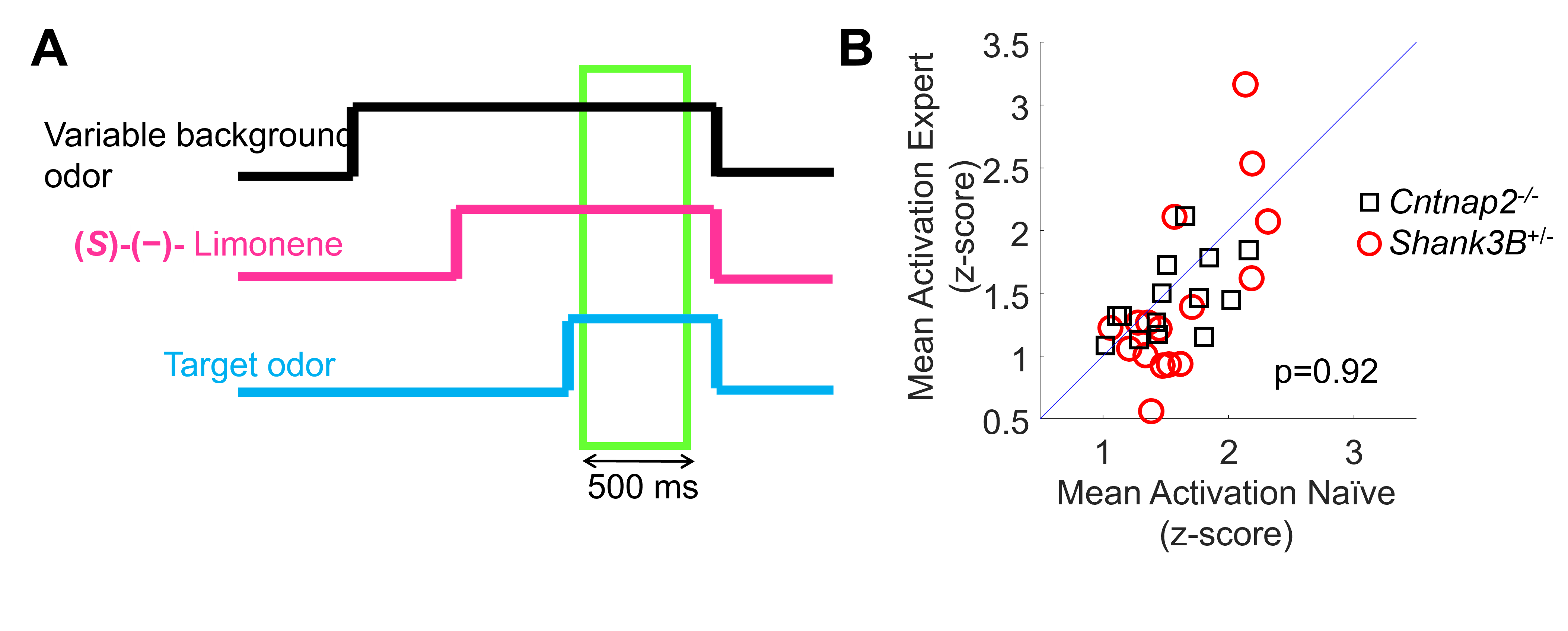

Supplement: Figure 6-1 — Target odor responses were not reduced as Shank3B+/- and Cntnap2-/- mice transition from being naïve to being experts. A. Target responses were quantified in a 500 ms window following the target onset. B. Mean average glomerular responses for target and background odors were not reduced with experience in Shank3B+/- and Cntnap2-/- mice. Download Figure 6-1, TIF file. [file eneuro-12-ENEURO.0271-25.2025-s003.tif]

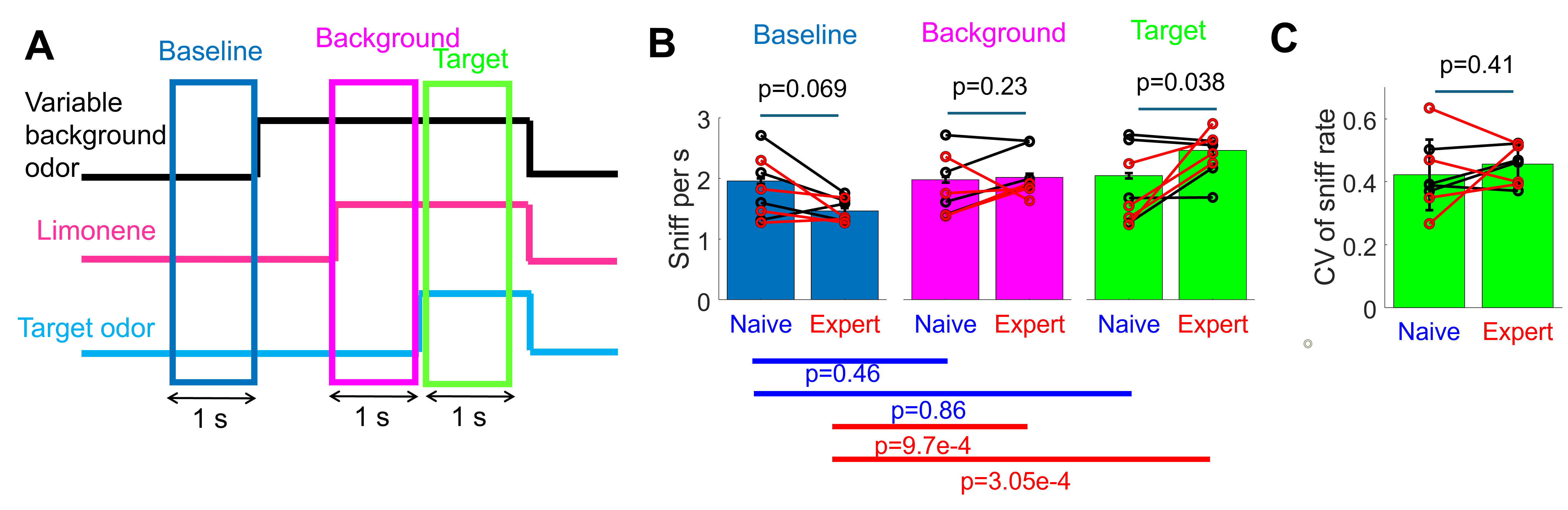

Supplement: Figure 6-2 — Sniffing responses to background odors did not change with experience in Shank3B+/- and Cntnap2-/- mice. A. Sniff responses to the backgrounds were evaluated in a 1 s window preceding the onset of the target odor. The target and odor mixtures were evaluated in a 1 s window following the onset of target odor. B. There was not significant difference in the sniff rate during the background period in between naïve and expert condition in the Shank3B+/- (in red) and Cntnap2-/- mice (in black). Lines represent individual animals. There was a significant increase in sniffing during the target period for the expert condition. C. The variability in the sniffing was unchanged between the naïve and the expert condition for Shank3B+/- and Cntnap2-/- mice. Download Figure 6-2, TIF file. [file eneuro-12-ENEURO.0271-25.2025-s002.tif]

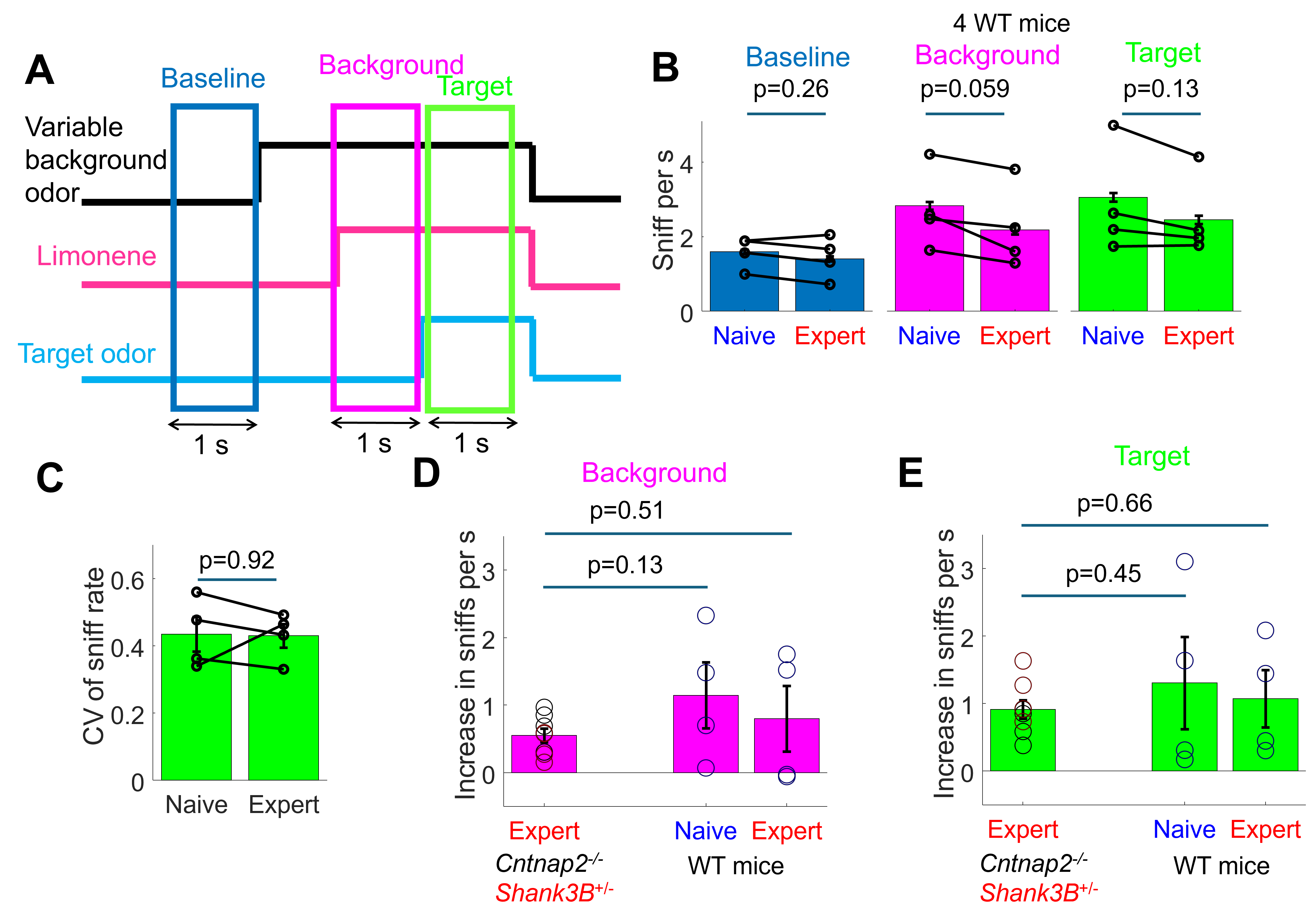

Supplement: Figure 6-3 — Sniffing responses to background odors did not change with experience in WT mice. A. Sniff responses to the backgrounds were evaluated in a 1 s window preceding the onset of the target odor. The target and odor mixtures were evaluated in a 1 s window following the onset of target odor. B. There was not significant difference in the sniff rate during the background period in between naïve and expert condition in the WT mice (in black). Lines represent individual animals. There was also no significant increase in sniffing during the target and odor period for the expert condition. C. The variability in the sniffing was unchanged between the naïve and the expert condition for WT mice. D-E. Naïve WT mice increased their sniff rate respect to the baseline for the background and target periods similar to expert mouse models of ASD. WT mice kept the increase in sniffing as experts. Download Figure 6-3, TIF file. [file eneuro-12-ENEURO.0271-25.2025-s001.tif]
